# Health care utilization and cost implications of Chile's 2024 nirsevimab strategy for RSV prevention: a counterfactual analysis

**DOI:** 10.1016/j.lana.2026.101475

**Published:** 2026-04-20

**Authors:** Denis Sauré, Amal Zgheib, Juan Pablo Torres, Marcel Goic, Charles Thraves, Jorge Pacheco, Javiera Burgos, Felipe Del Solar, Ignasi Neira, Miguel O'Ryan, Leonardo J. Basso

**Affiliations:** a Industrial Engineering Department, Universidad de Chile, Chile; b Instituto Sistemas Complejos de Ingeniería, Chile; c Departamento de Pediatría y cirugía Pediátrica, Facultad de Medicina, Universidad de Chile, Chile; d Departamento de Estadísticas e Información de Salud, Ministerio de Salud de Chile, Chile; e Instituto de Ciencias Biomédicas, Facultad de Medicina, Universidad de Chile, Chile

**Keywords:** RSV, Respiratory syncytial virus, Health care impact, Counterfactual analysis, Cost-benefit analysis, Synthetic control method

## Abstract

**Background:**

In 2024, Chile became the first Southern Hemisphere country to implement a universal nirsevimab immunization strategy against respiratory syncytial virus (RSV). This study evaluates the real-world health impact and cost-effectiveness of the program, specifically comparing seasonal (born April–September 2024) and catch-up (born October 2023–March 2024) cohorts.

**Methods:**

We performed a retrospective analysis using Chilean nationwide health registry data. The Augmented Synthetic Control Method (ASCM) utilized 2019–2024 data to estimate a counterfactual scenario for April–November 2024. Outcomes included outpatient medical attentions, basic/intermediate hospital bed-days, intensive care unit (ICU) bed-days, and days of maternal medical leave, identified through diagnoses for lower respiratory tract infections (LRTIs) and related viral agents. Differences between observed and predicted events were used to calculate treatment effects and cost-benefit ratios based on national healthcare costs.

**Findings:**

Nirsevimab was associated with substantial reductions across all metrics: approximately 20,430 days of medical leave, 25,620 medical attentions, 59,072 basic/medium bed-days, and 25,632 ICU bed-days. These reductions translated into a net economic benefit after subtracting the cost of immunization, of USD 23.50 million, including USD 15.50 million in the public healthcare sector. The catch-up cohort accounted for 53.41% of total cost savings, and the seasonal cohort for 46.59%.

**Interpretation:**

Chile's nationwide nirsevimab rollout substantially reduced infant RSV morbidity and healthcare utilization. These real-world findings demonstrate that universal immunization with long-acting monoclonal antibodies can significantly decrease hospitalizations and health system burden, supporting broad prevention strategies that include infants born prior to the RSV season.

**Funding:**

This research was partially funded by Instituto Sistemas Complejos de Ingeniería (ISCI) and by an independent research grant from Sanofi-AstraZeneca received by the ISCI team.

## Abstract (es)

**Antecedentes:**

Para la prevención del virus respiratorio sincicial (VRS), nirsevimab ha demostrado efectividad en condiciones reales en poblaciones generales de lactantes y se ha proyectado como una intervención con ahorro de costos cuando se implementa en estrategias estacionales con alta cobertura. Sin embargo, la evidencia sobre su impacto real en salud y su costo-beneficio es limitada. En 2024, Chile se convirtió en el primer país del hemisferio sur en implementar una estrategia universal de inmunización con nirsevimab. Nuestro objetivo fue evaluar el impacto sanitario real y la costo-efectividad de este programa a nivel nacional, así como analizar la contribución de dos grupos objetivo: los lactantes nacidos entre abril y septiembre de 2024 (cohorte estacional) y aquellos nacidos entre octubre de 2023 y marzo de 2024 (cohorte de rescate).

**Métodos:**

Se realizó un estudio retrospectivo utilizando registros nacionales de salud en Chile. Se empleó el Método de Control Sintético Aumentado (ASCM) con datos entre enero de 2019 y marzo de 2024 para estimar el escenario contrafactual en ausencia del programa entre abril y noviembre de 2024. Se evaluó el efecto en atenciones ambulatorias, días cama en unidades básicas/intermedias, días cama en unidades de cuidados intensivos (UCI) y días de licencia médica materna, identificados mediante diagnósticos de infecciones del tracto respiratorio inferior (ITRI) y agentes virales asociados. El número de eventos observados durante la temporada de VRS 2024 se comparó con las estimaciones contrafactuales. Los efectos del tratamiento se definieron como la diferencia entre ambos, y se analizaron desagregado por cohorte estacional y de rescate. Los costos fueron obtenidos del sistema nacional de salud, permitiendo un análisis de costo-beneficio en condiciones reales.

**Resultados:**

El programa de nirsevimab se asoció con reducciones sustanciales en todas las aristas: aproximadamente 20 430 días de licencia médica, 25 620 atenciones ambulatorias, 59 072 días cama en unidades básicas/intermedias y 25 632 días cama en UCI. Estas reducciones se tradujeron en un beneficio económico neto, tras descontar el costo de la inmunización, de USD 23.50 millones, incluyendo USD 15.50 millones en el sistema público. La cohorte de rescate generó el 53.41% del ahorro total, y la cohorte estacional el 46.59%.

**Interpretación:**

La implementación nacional de nirsevimab en Chile se asoció con una reducción importante de la morbilidad por VRS y del uso de servicios de salud en lactantes durante su primera temporada. Estos resultados en condiciones reales sugieren que la inmunización universal con un anticuerpo monoclonal de acción prolongada puede reducir de manera relevante las hospitalizaciones y la carga sobre el sistema de salud. Los hallazgos respaldan la implementación de estrategias amplias de prevención del VRS, incluyendo a los lactantes nacidos antes del inicio de la temporada.

**Financiamiento:**

Este estudio fue financiado parcialmente por el Instituto Sistemas Complejos de Ingeniería (ISCI) y por un fondo de investigación independiente de Sanofi-AstraZeneca adjudicado al equipo ISCI.

**Research in context**

**Evidence before this study**

We searched PubMed up to September 2025 using the terms “nirsevimab”, “respiratory syncytial virus”, “RSV”, “effectiveness”, “real-world”, and “impact”. Randomized trials and real-world studies consistently show that nirsevimab reduces RSV-related hospitalizations and intensive care admissions in infants. Phase 3 trials reported efficacy between 62% and 83% against RSV-associated lower respiratory tract infection. Observational studies from Europe and the USA found reductions of 66–90% in hospitalizations among infants in their first RSV season. Studies highlight the benefits of combining seasonal and catch-up strategies to protect infants born before and during the RSV season. Modeling and cost–benefit analyses indicate that broader prophylaxis strategies prevent more hospitalizations while remaining economically viable. However, no nationwide counterfactual evaluations of real-world impact had been conducted, particularly in the Southern Hemisphere, leaving a gap in evidence on population-level outcomes and economic impact outside clinical trial settings.

**Added value of this study**

This study provides a first nationwide counterfactual evaluation of nirsevimab impact, conducted in Chile during the 2024 RSV season following the first programme implementation in the Southern Hemisphere. Using a causal inference approach, we estimated counterfactual RSV-related outcomes in the absence of immunization with the Augmented Synthetic Control Method (ASCM). We assessed the days of medical leave saved, outpatient consultations and hospital bed use prevented (segregated by basic/medium and intensive care bed-days). In addition, we determined the impact separately for the seasonal and catch-up cohorts. Nirsevimab was associated with substantial reductions across all outcomes, leading in fact to a positive net benefit after costs, and with comparable benefits for both seasonal and catch-up groups.

**Implications of all the available evidence**

Our findings, together with previous trial evidence, show that immunization with nirsevimab for newborns during RSV season and infants less than 6 months before the season leads to substantial reductions across all outcomes, leading in fact to a positive net benefit after costs, and with comparable benefits for both seasonal and catch-up groups. These real-world results support the incorporation of nirsevimab into routine newborn-infant immunization schedules worldwide and the importance of considering a catch-up cohort. The reductions in hospitalizations and healthcare utilization confirm that benefits demonstrated in clinical trials can be achieved under routine conditions at a national scale. A universal strategy offers major health and economic gains, including fewer admissions and reduced seasonal pressure on health systems. By providing empirical, reliable data-modeling driven estimates of impact, this study can inform cost-benefit assessments and guide resource allocation for countries considering large-scale RSV immunization programmes.

## Introduction

Respiratory syncytial virus (RSV) is a leading cause of lower respiratory tract infection (LRTI) worldwide, particularly in infants and young children. Each winter, RSV epidemics place a heavy strain on health systems. In Chile, recent seasonal peaks have driven sharp increases in emergency visits and sustained high occupancy of pediatric wards and intensive care units (ICUs).^1^

Until 2023, national prophylaxis relied on palivizumab (AstraZeneca, Cambridge, UK), a monoclonal antibody targeting site II of the RSV fusion (F) protein. Because of its short half-life and high cost, palivizumab was reserved for a small group of high-risk infants, requiring monthly doses throughout the RSV season.^2^ In 2024, the Ministry of Health introduced a universal strategy based on nirsevimab (AstraZeneca, Cambridge, UK; Sanofi, Paris, France) through the National Immunization Programme, supported by retrospective cost-effectiveness analyses.^1^ Unlike palivizumab, nirsevimab has an extended half-life, enabling protection with a single intramuscular dose. Operational details of the campaign and its implementation have been reported previously.^2^

Evidence from randomized trials and observational studies shows that nirsevimab reduces RSV-related illness,^2^, ^3^, ^4^, ^5^, ^6^, ^7^, ^8^, ^9^, ^10^, ^11^, ^12^, ^13^, ^14^, ^15^ and cost-effectiveness studies have projected substantial reductions in healthcare and cost-saving in seasonal strategies with high coverage.^1^^,^^16^, ^17^, ^18^ However, its real-world impact at the national scale—particularly whether universal campaigns reduce pressure on health services during seasonal epidemics—remains uncertain. Addressing this question requires moving beyond trial settings to population-level evaluations under routine conditions. A fundamental methodological challenge is estimating the counterfactual: what healthcare demand would have occurred without the campaign. Simple before–and–after or cross-sectional comparisons risk entangling intervention effects with seasonal variation or unrelated system changes.

We aimed to address this gap using robust causal inference methods.^19^, ^20^, ^21^, ^22^ This method allows to construct the hypothetical scenario that would have occurred without the intervention, by carefully selecting a finite number of characteristics from a diverse set of explanatory variables and temporal components, related with potential immunization effect, to find the best weights for the control units that together, replicate with high concordance, the RSV epidemiological curve during the previous years. We compared the observed vs the contrafactual number of events for four outcomes: parental medical-leave (ML) days, outpatient visits, basic/intermediate pediatric hospital bed-days, and ICU bed-days. By quantifying the differential impact of both scenarios (with and without intervention) on these four healthcare outcomes, and obtaining the mean cost for each using national health care system information, we determined the direct health care and economic impact of the 2024 immunization strategy with coverage of 94.11%.^2^

## Methods

### Study setting and intervention

Chile's national RSV prophylaxis strategy with nirsevimab was implemented on April 1, 2024, approximately one month before the anticipated RSV season, and concluded on September 30, 2024. The campaign targeted three groups: (i) infants born between April and September 2024 (seasonal cohort); (ii) infants born between October 2023 and March 2024 (catch-up cohort); and (iii) infants with high-risk conditions previously eligible for palivizumab (high-risk cohort). High-risk infants not included in the seasonal or catch-up cohorts were excluded because of small numbers. Immunization coverage across the target cohorts reached 94.11%, corresponding to 91.2% in the catch-up cohort and 97.1% in the seasonal cohort. Further details of the campaign and its implementation are reported by Torres et al. (2025).^2^

### Study period

The analysis covered January 1, 2019, to November 30, 2024. Although the official campaign began on April 1, 2024, registry data indicated that the first administration of nirsevimab occurred March 25, 2024. To minimize anticipation effects, the intervention date was set at March 31, 2024. Follow-up was extended through November to capture the full seasonal effect.

### Data sources

We used four nationwide registries routinely maintained by the Ministry of Health: MLs, inpatient care, outpatient care, and respiratory virus circulation (see Appendix p 1). Datasets were anonymized before analysis. Records lacking diagnostic information, rejected claims, or admissions for patients older than 15 years without respiratory diagnoses were excluded. Additional consistency checks were performed to identify duplicate entries and implausible dates in the records. Outpatient data were available only for the public sector. To harmonize across sources and account for seasonality, records were aggregated at the weekly level (see Appendix p 2).

### Outcomes

Since RSV is often under-coded, we considered a broader category of diagnoses for lower respiratory tract infections (LRTIs) and related viral agents, not only RSV-related diagnosis (see Appendix p 3) to estimate the global impact. This includes B95–B98, J09–J18, J20–J22. Primary outcomes in infants under one year were: (i) parental medial leave days (ML), (ii) outpatient visits (MA), (iii) basic/intermediate bed-days (BM), and (iv) ICU bed-days (see Appendix pp 4–6).

### Statistical analysis

We used the Augmented Synthetic Control Method (ASCM)^19^ to estimate counterfactual series (see Appendix pp 7–9), after assessing several variants of similar methods during the 2022 and 2023 winter seasons (see Appendix p 10). The ASCM constructs a synthetic control as a weighted combination of other unaffected-time series (the donor pool) that reproduces pre-treatment trajectories of the variables of interest. The method then uses this synthetic control to estimate the counterfactual behavior in the post-treatment periods. The weights are selected through an optimization problem to minimize the quadratic difference between the original series and the synthetic control in the pre-treatment periods. The donor pool was formed by units defined as age–disease groups unlikely to be affected by the campaign and that in a combined analysis best replicated the RSV epidemiological curve of preintervention years, following prior literature,^20^ and supplemented with outpatient and surveillance data. Children aged 1–4 years with LRTI were excluded to avoid spillover effects (see Appendix pp 11–12).

Dummy controls units captured month and holiday effects, and covariates were selected if they improved pre-intervention fit. Outcomes and covariates were normalized over the pre-intervention period to ensure comparability across heterogeneous sources^21^^,^^22^ (see Appendix pp 13–15). We recovered the five control units with the highest absolute weights to assess their contribution to pre-intervention fit. Pre-intervention mean error (ME) was used to assess the model fit. Treatment effects for each outcome were defined as the difference between the observed number of events during the 2024 RSV season and the hypothetical number of events calculated using the synthetic series. Significance of the treatment effect was assessed using placebo tests in time with intervention dates reassigned in 2022–23 (see Appendix pp 16–17).

Cumulative effects were defined as the aggregate difference between observed and synthetic series over 35 weeks post-intervention. The 95% confidence intervals (CIs) were derived from the distribution of placebo residuals (see Appendix p 18). Effects were translated into avoided ML days, outpatient visits, and hospital bed-days, disaggregated by health sector (private vs public).

We further decomposed effects by Immunization cohort using Monte Carlo simulations based on coverage and effectiveness estimates,^2^ and present 95% confidence intervals (see Appendix pp 19–20). For MLs, all reductions were attributed to the catch-up cohort, since mothers of infants in the seasonal cohort remained covered by statutory postnatal leave.

### Economic evaluation

Unit costs for outpatient visits and bed-days were obtained from public sector estimates^1^; values likely underestimate system-wide savings. The replacement of palivizumab by nirsevimab for eligible groups in 2024 was estimated had a direct cost reduction of USD$10.79 million. The total cost of the 2024 nirsevimab campaign was USD$45.28 million (see Appendix p 21). ML costs were valued at approximately USD$42 per day,^23^^,^^24^ all attributable to the public sector spending.

### Role of the funding source

The funders had no role in the design, conduct, analysis, or reporting of this study, nor in the decision to submit for publication.

### Ethics and guidelines

The Chilean government routinely collects the data used for this study. Ethical approval was not required because the routine immunization and data collection process was not affected by the realization of this study. Approval for the use of the data, assuring anonymity, was granted by the Minister of Health (MoH), whose rigorous ethical standards in data collection and commitment to maintaining data integrity and anonymity obviated the need for additional ethical approval.

## Results

### Model fit and treatment effects

**Fig. 1:**
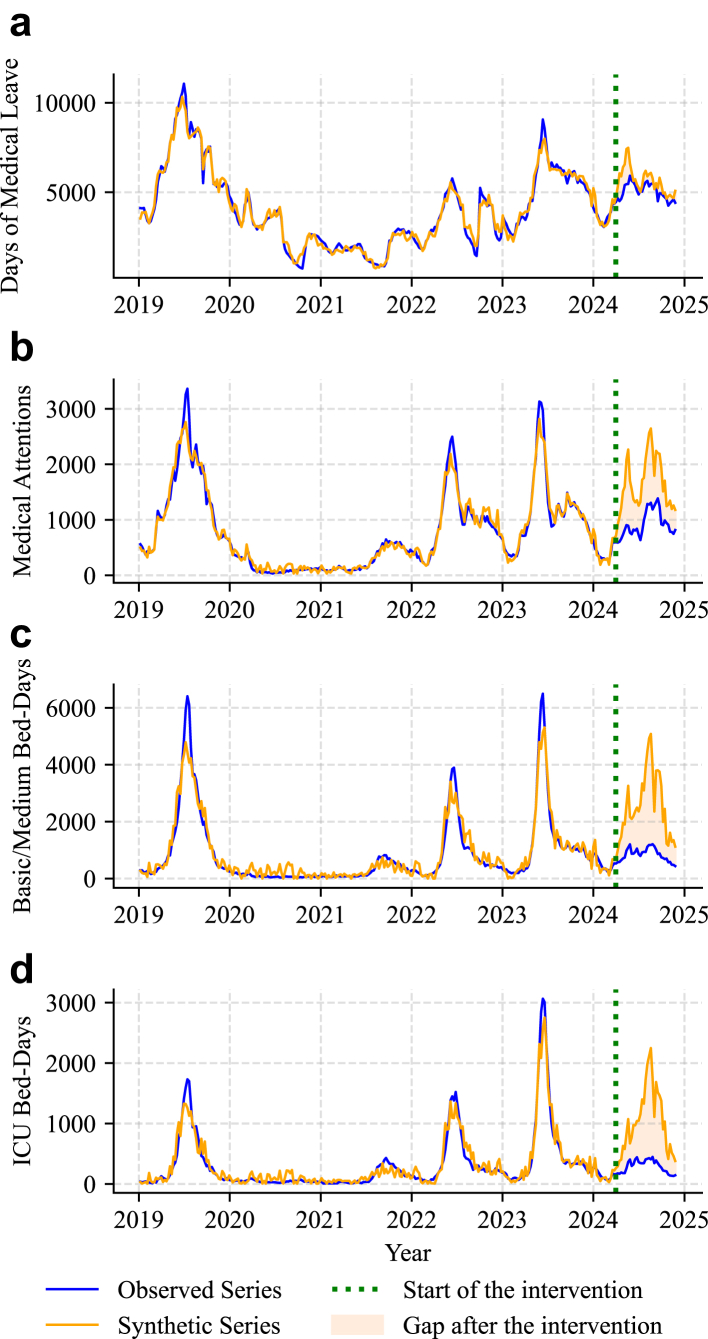
**Observed and estimated outcome series (LRTIs and related viral agents, for infants under 1 year of age).** (a) ML: medical leave; (b) MA: medical attention; (c) BM: basic and medium bed-day; (d) ICU: intensive care unit bed-day.

Synthetic control estimates closely reproduced the observed series during the pre-intervention period and diverged upward thereafter, indicating reductions in outcomes attributable to the campaign (Fig. 1). Top five weights for control units contributing to each outcome are reported in Table 1 (see Appendix pp 22–25). For example, respiratory diseases unrelated to RSV in infants under 1 year and RSV-positive cases in adults aged 65 years or older were among the most influential controls.

**Table 1:** Top five control units used by the augmented synthetic control method.

Unit	Control unit	Age group	Units	Weight
ML	Respiratory Diseases Non-Related to RSV	<1	Days of medical leave	0.25
	Respiratory Diseases Non-Related to RSV	<1	Basic/medium bed-days	0.24
	Eye and ear diseases	<1	Days of medical leave	0.14
	LRTI and Related Agents	65+	Medical attentions	0.09
	RSV	65+	Bed-days in surveillance hospitals	0.06
MA	RSV	65+	Bed-days in surveillance hospitals	0.31
	LRTI and Related Agents	65+	Medical attentions	0.24
	Respiratory Diseases Non-Related to RSV	1–4	Medical attentions	0.21
	Respiratory Diseases Non-Related to RSV	<1	Basic/medium bed-days	0.17
	LRTI and Related Agents	5–14	Medical attentions	0.11
BM	RSV	65+	Bed-days in surveillance hospitals	0.55
	Respiratory Diseases Non-Related to RSV	<1	Basic/medium bed-days	0.26
	Adenovirus	All	Positive cases in healthcare surveillance centers	0.13
	Influenza B	All	Positive cases in healthcare surveillance centers	0.09
	LRTI and Related Agents	65+	Medical attentions	0.06
ICU	RSV	65+	Bed-days in surveillance hospitals	0.51
	Adenovirus	All	Positive cases in healthcare surveillance centers	0.28
	Respiratory Diseases Non-Related to RSV	<1	Basic/medium bed-days	0.12
	Metapneumovirus	All	Positive cases in healthcare surveillance centers	0.09
	Winter Holidays	–	–	0.05

ML: medical leave; MA: medical attention; BM: basic and medium bed-day; ICU: intensive care unit bed-day.

Pre-intervention model fit was high across outcomes, and post/pre-treatment differences were significant for three of the four outcomes evaluated. For ML, the pre-treatment mean error (ME) was 2.11, with a post/pre-treatment ME difference of −623.95 (p = 0.21). For medical attentions (MA), pre-treatment ME was −2.92 with a post/pre-treatment ME difference of −681.58 (p = 0.03). For basic/medium bed-days (BM), pre-treatment ME was −31.35 with a post/pre-treatment ME difference of −1650.88 (p = 0.03). For ICU bed-days, pre-treatment ME was −8.61 with a post/pre-treatment ME difference of −713.57 (p = 0.03) (Table 2).

**Table 2:** Fitness metrics, p-values, and saving estimates for each outcome (LRTIs and related viral agents, for infants under 1 year of age).

Outcome	ML	MA	BM	ICU
Pre-Treatment ME	2.11	−2.92	−31.35	−8.61
Post/Pre-Treatment ME Difference	−623.95	−681.58	−1650.88	−713.57
P-value	0.21	0.03	0.03	0.03
Total Savings (95% CI)	20,429.92 (1346.97–19,245.39)	25,619.74 (23,046.7–26,932.42)	59,072.26 (48,469.57–59,836.59)	25,632.14 (24,415.79–31,141.04)
Public Savings	16,546.34	18,622.34	49,031.55	18,385.59
Catch-up Savings Percentage (95% CI)	100.00% (−)	53.27% (44.64%–64.15%)	49.73% (40.64%–61.33%)	55.25% (41.58%–71.64%)
Seasonal Savings Percentage (95% CI)	0.00% (−)	46.73% (35.85%–55.36%)	50.27% (38.67%–59.36%)	44.75% (28.36%–58.42%)

ML: medical leave; MA: medical attention; BM: basic and medium bed-day; ICU: intensive care unit bed-day.

### Health care impact

The counterfactual analysis estimated that, over the 2024 season, the campaign prevented overall 20,429.92 days of ML (95% CI 1346.97–19,245.39), 25,619.74 medical attentions (95% CI 23,046.7–26,932.42), 59,072.26 basic/medium bed-days (95% CI 48,469.57–59,836.59), and 25,632.14 ICU bed-days (95% CI 24,415.79–31,141.04). Specifically, within the public sector, reductions included 18,622.34 outpatient visits, 49,031.55 basic/medium bed-days, and 18,385.59 ICU bed-days; all ML savings were attributable to the public system.

### Cohort-specific contributions

**Fig. 2:**
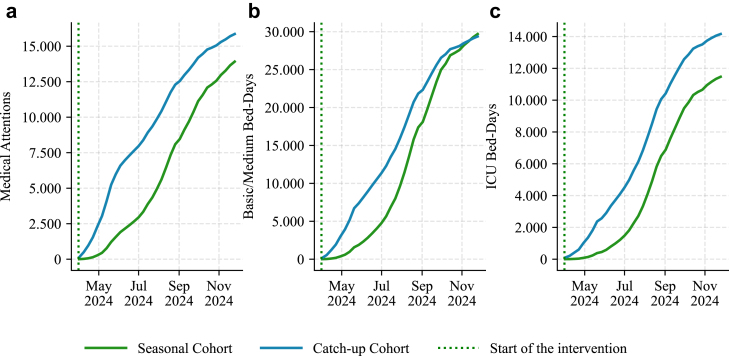
**Cumulative savings by cohort group, for each outcome (LRTI and related viral agents, for infants under 1 year of age).** (a) MA: medical attention; (b) BM: basic and medium bed-day; (c) ICU: intensive care unit bed-day.

Cohort segregation indicated that, among outpatient visits, 53.27% (95% CI 44.64–64.15) of the reduction was attributable to the catch-up cohort and 46.73% (95% CI 35.85–55.36) to the seasonal cohort. For basic/intermediate bed-days, 49.73% (95% CI 40.64–61.33) of savings were generated by the catch-up cohort and 50.27% (95% CI 38.67–59.36) by the seasonal cohort. For ICU bed-days, 55.25% (95% CI 41.58–71.64) of reductions were attributed to the catch-up cohort and 44.75% (95% CI 28.36–58.42) to the seasonal cohort. Temporal dynamics showed that cumulative reductions in outpatient visits, bed occupancy were mainly driven by the catch-up cohort at the beginning of the campaign, with the seasonal cohort catching-up later (Fig. 2).

### Economic evaluation

The 2024 nirsevimab programme cost USD$45.28 million, while USD$10.79 million were saved by avoiding palivizumab use in high-risk infants. Estimated healthcare savings amounted to USD$68.78 million (95% CI 60.23–69.23), of which 75.91% were attributable to reductions in inpatient care, yielding a net benefit of USD$23.50 million (95% CI 14.95–23.95) (Table 3). From the public system perspective, savings reached USD$60.78 million, with a net benefit of USD$15.50 million.

**Table 3:** Overall savings and net benefit estimates.

Costs		
Nirsevimab	Costs of nirsevimab program in 2024.	$45.28 M

Savings			
Outcome	Description	Total (95% CI)	Public
ML	Days of medical leave.	$0.86 M ($0.06 M–$0.81 M)	$0.86 M
MA	Medical attention.	$4.92 M ($4.42 M–$5.17 M)	$4.23 M
BM	Basic/medium bed-days.	$24.46 M ($20.07 M–$24.77 M)	$21.03 M
ICU	ICU bed-days.	$27.76 M ($26.44 M–$33.73 M)	$23.87 M
Palivizumab	Avoided use of palivizumab in 2024.	$10.79 M	$10.79 M
Total savings		$68.78 M ($60.23 M–$69.23 M)	$60.78 M

Net benefit		
Group	Total (95% CI)	Public
Seasonal total net benefit percentage	46.59% (34.41%–56.87%)	–
Catch-up total net benefit percentage	53.41% (43.13%–65.59%)	–
Total net benefit	$23.50 M ($14.95 M–$23.95 M)	$15.50 M

ML: medical leave; MA: medical attention; BM: basic and medium bed-day; ICU: intensive care unit bed-day.

The largest components of savings came from avoided ICU bed-days accounting for 40.36% of savings (USD$27.76 million, 95% CI 26.44–33.73) and basic/medium bed-days accounting for 35.56% of savings (USD$24.46 million, 95% CI 20.07–24.77). Additional contributions came from reduced outpatient visits (USD$4.92 million, 95% CI 4.42–5.17) and (possibly) ML (USD$0.86 million, 95% CI 0.06–0.81). When disaggregated by Immunization cohort, the catch-up group contributed 53.41% (95% CI 43.13–65.59) of total net benefits and the seasonal cohort 46.59% (95% CI 34.41–56.87).

## Discussion

Our findings indicate that Chile's 2024 nationwide rollout of nirsevimab was associated with substantial reductions across four direct health care outcomes, with the strongest effects observed in hospital-based indicators. The robustness of the synthetic control estimates was supported by the high resemblance between the 2024 RSV epidemiological curve and the synthetic curve constructed after analysis. This is reflected in the distribution of donor weights. Across outcomes, the largest weights were systematically assigned to series with close epidemiological or demographic similarities to the treated units, indicating that the method consistently selected biologically plausible comparators rather than unrelated conditions. For ML, the top contributors were the series of medical leave days and basic/medium bed-days caused by respiratory diseases non-related to RSV in infants under one year of age, reflecting clinically credible pathways of disease transmission and severity. For the other three outcomes, the number of bed-days caused by RSV in surveillance hospitals for people above 65 years of age consistently emerged as the dominant donor series, highlighting its role as an indicator of seasonal RSV circulation and severity. In the case of BM and ICU, both series of adenovirus surveillance and basic/medium bed-days caused by respiratory diseases non-related to RSV in infants under one year of age were in the top three-largest weights assigned. This alignment between statistical weight allocation and established epidemiological patterns strengthens confidence in the validity and substantive relevance of the counterfactual trajectories, consistent with prior applications of synthetic control methods in epidemiological settings.^20^, ^21^, ^22^

Specifically, we estimated reductions of 20,430 days of ML, 25,620 outpatient attentions, 59,072 basic/intermediate bed-days, and 25,632 ICU bed-days. For days of ML, the effect was not statistically significant (p-value of 0.21); however, considering that a total of 507,339 days were granted in 2023 and 483,326 in 2024,^25^ the magnitude of the estimated decrease was consistent with year-to-year fluctuations in administrative data. In contrast, all other outcomes showed statistically significant reductions. The concentration of effects in hospital-based outcomes is especially relevant, as these represent the most resource-intensive components of the healthcare system. These findings are consistent with a growing body of real-world evidence showing substantial reductions in RSV-related hospitalizations and severe outcomes following nirsevimab implementation across multiple settings.^3^, ^4^, ^5^, ^6^, ^7^, ^8^, ^9^, ^10^, ^11^^,^^14^

Public sector savings accounted for approximately 22,032 avoided medical attentions, 50,802 avoided basic/intermediate bed-days, and 22,044 avoided ICU bed-days. These results highlight the critical contribution of the intervention in alleviating pressure on public-sector capacity, which carries the greatest burden of severe respiratory morbidity, while simultaneously producing measurable, though smaller, reductions in private-sector demand.

Across all treated units, the pre-treatment mean errors (MEs) were small in magnitude, reflecting a high-quality pre-intervention fit. In contrast, the differences between post-treatment and pre-treatment MEs were consistently negative and of much larger magnitude than the corresponding pre-treatment Mes, reinforcing the interpretation that deviations observed after the intervention reflected genuine treatment effects.

The disaggregation by cohort revealed complementary and time-dependent dynamics. The catch-up cohort, although immunized at slightly lower coverage levels than the seasonal group, comprised the majority of the eligible infant population at the beginning of the season. This positioning generated early-season protection and accounted for most of the avoided cases in the initial epidemic period. Specifically, the catch-up group contributed 55.25% of avoided ICU bed-days and nearly half of the savings in basic/medium bed-days (49.73%) and in medical attentions (52.27%). The cumulative distributions corroborate this front-loaded effect: between May and September 2024, the catch-up cohort dominated the reductions in medical attentions, basic/intermediate and ICU bed-days, reflecting its critical role in mitigating the epidemic peak. In contrast, the seasonal cohort progressively increased its contribution as births accumulated and coverage expanded. By the end of the intervention period, this cohort accounted for 50.27% avoided basic/intermediate bed-days, 46.73% avoided outpatient consults, and 44.75% avoided ICU bed-days. Together, these findings demonstrate the strategic value of combining catch-up and seasonal protection (in line with recent evidence highlighting the complementary benefits of at-birth and catch-up Immunization strategies^5^): the former provides rapid, front-loaded reductions in severe morbidity, while the latter sustains population-level protection throughout the epidemic curve, ensuring that benefits extend beyond the initial surge.

From an economic perspective, the intervention generated estimated savings –in the same year of the campaign– of USD$68.78 million, of which approximately USD$60.78 million occurred in the public sector. More than 90% of these savings were concentrated in hospital care, with basic/intermediate and ICU bed-days each contributing approximately USD$24 million and USD$28 million, respectively, which is consistent with prior cost-effectiveness analyses.^16^, ^17^, ^18^ While the absolute reduction was highest for ICU bed-days, the combination of basic/intermediate and ICU bed-days savings accounted for the largest economic impact, reflecting both the volume of hospitalizations prevented and the high unit cost of intensive care. The cost savings in outpatient medical consults (USD$4.92 million) and days of ML (USD$0.86 million), smaller in absolute magnitude, nonetheless illustrate the broad impact of the program across the continuum of care. The 95% confidence intervals confirmed that these estimates were statistically robust, with narrower bounds for outpatient outcomes and wider intervals for hospital-based measures, consistent with the greater variability of severe cases.

The distribution of cost savings between cohorts mirrored their clinical contributions. Monte Carlo simulations showed that the catch-up cohort accounted for 53.41% of total savings, compared with 46.59% for the seasonal cohort. For the catch-up cohort, the estimated savings share ranged from 43.13% to 65.59%, while for the seasonal cohort it ranged from 34.41% to 56.87%. These overlapping intervals suggested that, although the catch-up strategy initially dominated in generating savings, both cohorts made substantial and statistically comparable contributions over the course of the season. Importantly, the catch-up strategy ensured an immediate reduction in expensive outcomes at the beginning of the season, while the seasonal strategy sustained cost savings as coverage expanded.

Finally, when accounting for the program cost (USD$45.28 million), the net benefit of the 2024 campaign was estimated at USD$23.50 million. This balance highlights the fiscal value of the strategy aligns with previous modeling studies suggesting that high-coverage nirsevimab strategies can be cost-saving or cost-effective at the population level^16^, ^17^, ^18^: despite substantial upfront investment for the nirsevimab strategy, the reduction in healthcare utilization and the discontinuation of palivizumab generated a clear and positive return within the same year. Approximately USD$15.50 millions of this net benefit accrued to the public sector, reinforcing the program's importance in strengthening the sustainability of Chile's national health system. Note that without the inclusion of the catch-up, the net benefit would decrease from $23.5 M to approximately $10.95 M.

Our results provide robust evidence that Chile's 2024 nirsevimab campaign substantially reduced LRTIs morbidity and yielded a positive net economic benefit under real-world conditions. The combined use of catch-up and seasonal immunization was essential to achieve these outcomes. By simultaneously alleviating pressure on hospital capacity, reducing outpatient demand, and generating significant fiscal savings, the intervention not only strengthened pediatric resources in Chile but also offered a scalable model for other countries evaluating the nationwide adoption of nirsevimab, complementing emerging international real-world evidence from Europe and North America.^3^, ^4^, ^5^, ^6^, ^7^, ^8^, ^9^^,^^14^

### Limitations

Our study has several limitations. First, although the ASCM provides a robust counterfactual framework, it depends on the quality and completeness of pre-intervention data.^19^, ^20^, ^21^, ^22^ Any unmeasured confounding or structural changes unrelated to the intervention could bias the estimated effects. For example, variations in hospital resource availability and admission practices over time could lead to underestimation of the intervention's impact, whereas demographic shifts, such as population aging, could result in overestimation. Additionally, changes in assistance behavior may have influenced the estimates, although the direction of this potential bias is uncertain. Second, the outcomes were derived from administrative data, which may be subject to misclassification, reporting delays, or underreporting. In particular, variability in coding practices across health facilities and outcomes could influence accuracy, for example, in the classification of medical attentions. Additionally, as the study only included aggregated public sector emergency visits (medical attentions), we were unable to assess the short- or mid-term morbidity impact or primary healthcare utilization related to RSV infection. Third, while the study design aimed to isolate the effect of the national nirsevimab program, seasonal variations in RSV circulation or changes in healthcare-seeking behavior during the study period may have contributed to the observed reductions. These external influences cannot be fully disentangled from the estimated program effects. Fourth, the economic evaluation was based on public costs, which may underestimate the broader economic value of the intervention; yet, using average value from the Superintendencia de Salud (the regulatory body), a ‘back of the envelope’ calculation hints that the $8 M in net benefit that the private sector brings would roughly increase to $10.41. Fifth, indirect societal costs (e.g., caregiver burden, productivity losses), as well as potential long-term respiratory morbidity related to RSV infection, were not included, which could further underestimate the overall economic impact.

### Conclusions

Our nationwide analysis demonstrates that the campaign substantially reduced LRTIs morbidity and healthcare utilization, with the largest savings concentrated in hospital-based outcomes: reductions of 59,072 basic/intermediate bed-days and 25,632 ICU bed-days. Importantly, disaggregation by cohort revealed the complementary contributions of the catch-up and seasonal groups: the catch-up cohort delivered early-season reductions, particularly in severe outcomes such as ICU bed-days (55.25%), while the seasonal cohort progressively contributed to sustained savings as coverage expanded. This cohort-specific analysis highlighted not only the clinical relevance of combining strategies but also their joint contribution to maximizing the population-level impact.

The intervention generated direct savings of approximately USD$68.78 million, of which the catch-up cohort contributed 53.41%, while the seasonal cohort contributed 46.59%, underscoring the importance of both strategies in achieving population-wide protection. When accounting for program implementation costs, the campaign produced a net benefit of USD$23.50 million (USD$15.50 million in the public sector).

Overall, these results provide rigorous real-world evidence that the 2024 nirsevimab rollout in Chile delivered measurable reductions in LRTIs morbidity, alleviated hospital burden, and produced a clear net monetary benefit, demonstrating the potential of expanding immunization to protect an even larger number of children in future seasons. The findings are consistent with both clinical trial efficacy and accumulating real-world effectiveness evidence across diverse health systems,^3^, ^4^, ^5^, ^6^, ^7^, ^8^, ^9^, ^10^, ^11^^,^^14^ and underscore the value of ASCM as a tool for policy evaluation and support the integration of catch-up and seasonal immunization strategies as an effective approach to controlling RSV epidemics.

## Contributors

DS, JPT, MO’R, JP, and LJB conceived the study. LJB led and coordinated the project. JP and JB directed the DEIS team that worked on data consolidation and anonymization. LJB and DS directed the ISCI team that performed the statistical analysis. DS, MG, CT, AZ, FD, and IN were responsible for data cleaning, analyses, and generating tables and figures. DS, AZ, and LJB drafted the original manuscript. All authors contributed to the methods, revised the manuscript, and approved the final version. LJB, DS, JP, and JB have accessed and verified the data. All authors had full access to all the data in the study and had final responsibility for the decision to submit for publication.

## Data sharing statement

Anonymized data sets were shared under a formal collaboration agreement between Chile's Ministry of Health and ISCI. Data was anonymized by DEIS officials to comply with current legislation, ensuring confidentiality, and then stored on servers at ISCI. Aggregate data is openly accessible either on the DEIS website or within the repositories of Chile's National Statistics Institute. Data cannot be shared directly by the authors because of data protection regulations. Data is accessible to authorized researchers after an application to the Ministry of Health of Chile.

## Declaration of interests

DS, JPT, MO’R and LJB received an unrestricted grant from Sanofi for analysis support. JPT reported grants from Sanofi unrelated to the current study. No other disclosures were reported.
