# Supplementary material for: Health care utilization and cost implications of Chile's 2024 nirsevimab strategy for RSV prevention: a counterfactual analysis
Source: Lancet Reg Health Am. 2026 Apr 20;59:101475. doi: 10.1016/j.lana.2026.101475 (PMC13122228; doi:10.1016/j.lana.2026.101475)
Supplement: Supplementary Material [file mmc1.docx]

**Supplementary Material**

**Health Care Utilisation and Cost Implications of Chile’s 2024 Nirsevimab Strategy for RSV Prevention: a counterfactual analysis**

Denis Sauré, Amal Zgheib, Juan Pablo Torres, Marcel Goic, Charles Thraves, Jorge Pacheco, Javiera Burgos,

Felipe Del Solar, Ignasi Neira, Miguel O’Ryan, Leonardo J. Basso

**Sentinel Hospitals in ILI/SARI Surveillance**

Respiratory virus circulation data were retrieved from the national surveillance system managed by the Epidemiology's Department of Ministry of Health. These reports included weekly counts of ambulatory consultations and hospital admissions for influenza-like illness (ILI) and hospital admissions for severe acute respiratory infection (SARI) from a network of six sentinel hospitals (see Table S2). Counts were aggregated weekly by virus type and age group as reported by the surveillance program.

| **ID** | **Sentinel hospital (location)** |
| --- | --- |
| 1 | Hospital Dr. Hernán Henríquez Aravena (Temuco) |
| 2 | Hospital de Puerto Montt |
| 3 | Hospital Clínico Regional Dr. Guillermo Grant Benavente (Concepción) |
| 4 | Hospital San Juan de Dios (Santiago) |
| 5 | Hospital Dr. Gustavo Fricke (Viña del Mar) |
| 6 | Hospital Dr. Ernesto Torres Galdames (Iquique) |

**Table S1.** Sentinel hospitals of the national surveillance system.

**Construction of Daily Time Series**

Basic/intermediate and ICU Daily Bed Occupancy

Each hospitalization record included the dates of admission and discharge, ICD-10 diagnosis, hospital, trajectory across different levels of care (bed type), and length of stay.

A bed was considered occupied if the patient remained for at least one night. Let [
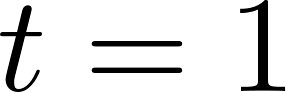
](https://www.codecogs.com/eqnedit.php?latex=t%3D1#0) denote the day of admission to level [
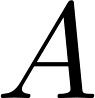
](https://www.codecogs.com/eqnedit.php?latex=A#0) and [
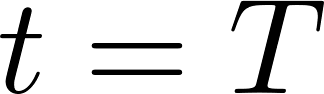
](https://www.codecogs.com/eqnedit.php?latex=t%3DT#0) the discharge or transfer date. The length of stay in care level A was computed as [
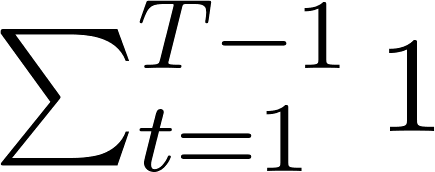
](https://www.codecogs.com/eqnedit.php?latex=%5Csum_%7Bt%3D1%7D%5E%7BT-1%7D%201#0), represented as [
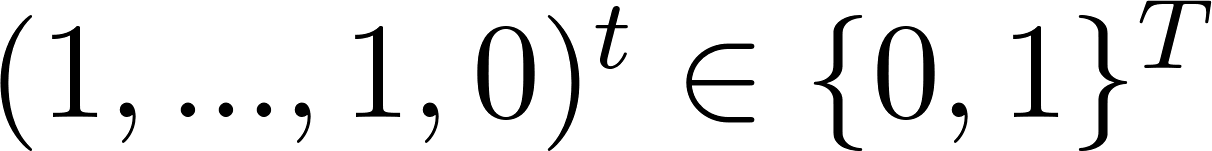
](https://www.codecogs.com/eqnedit.php?latex=(1%2C...%2C%201%2C%200)%5Et%20%5Cin%20%5C%7B0%2C1%5C%7D%5ET#0), i.e., each night spent was counted as one occupied bed-day.

Days of Medical Leave

Each medical leave record included the start and end dates, ICD-10 diagnostic category, and region.

For each individual, a time series was constructed by assigning one unit per day covered by the medical leave. Formally, let [
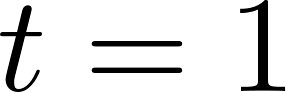
](https://www.codecogs.com/eqnedit.php?latex=t%3D1#0) denote the first day of the leave and [
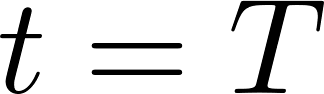
](https://www.codecogs.com/eqnedit.php?latex=t%3DT#0) the last day. The total number of days was [
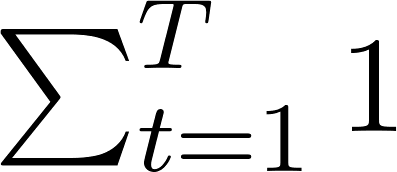
](https://www.codecogs.com/eqnedit.php?latex=%5Csum_%7Bt%3D1%7D%5E%7BT%7D%201#0), represented in the time series as [
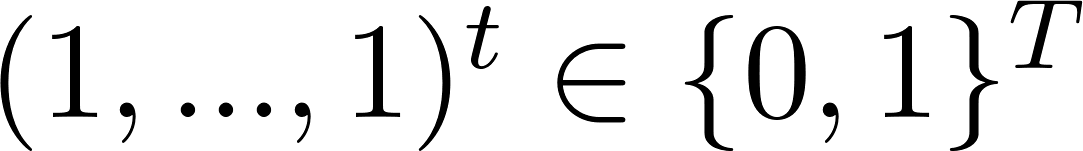
](https://www.codecogs.com/eqnedit.php?latex=(1%2C...%2C%201)%5Et%20%5Cin%20%5C%7B0%2C1%5C%7D%5ET#0).

Medical Attentions

Daily series of medical attentions were derived directly from the outpatient dataset, under the assumption that each attention corresponded to a single day.

**Treated Units**

LRTIs and related agents outcomes were identified using broader groups of ICD-10 codes tailored to each data source.

Medical leaves and hospital admissions:

- B95–B98: Bacterial and viral infectious agents.
- J09–J18: Influenza and pneumonia.
- J20–J22: Other acute lower respiratory infections.

Medical attentions:

- J09–J11: Influenza.
- J12–J18: Pneumonia.
- J20–J21: Acute bronchitis and bronchiolitis.
- J22, J30–J39, J47, J60–J98: Other respiratory causes.

This broader grouping was necessary because diagnostic classifications varied across datasets and restricting the analysis to RSV-coded cases alone would likely underestimate the true impact of the intervention. In practice, the clinical manifestations of RSV infection often overlap with those of other respiratory viruses (e.g., influenza, parainfluenza, or metapneumovirus).

By aggregating outcomes into the clinically relevant category of LRTIs and related agents, the analysis captured both the direct effect on confirmed RSV cases and the indirect effect on conditions where RSV was a major causal agent but not explicitly recorded in administrative data. This approach provided a more comprehensive and realistic estimate of the population-level impact of the immunization strategy.

**Outcomes**

The time series of treated LRTIs and Related Agents units showed pronounced seasonal fluctuations across outcomes, with strong epidemic peaks in 2019–2020 and again in 2023, while the disruption caused by COVID-19 is evident in 2021–2022 (see Figure S1). ML and MA account for the largest absolute volumes, whereas hospital-related outcomes (BM and ICU) present sharper but narrower surges concentrated in epidemic weeks, underscoring the different ways in which outpatient and inpatient indicators capture disease burden. Yearly profiles reveal a notable decrease in 2024, when both weekly peaks and cumulative totals fall below prior levels (see Figure S2). Summarized characteristics of outcomes are shown in Table S2.


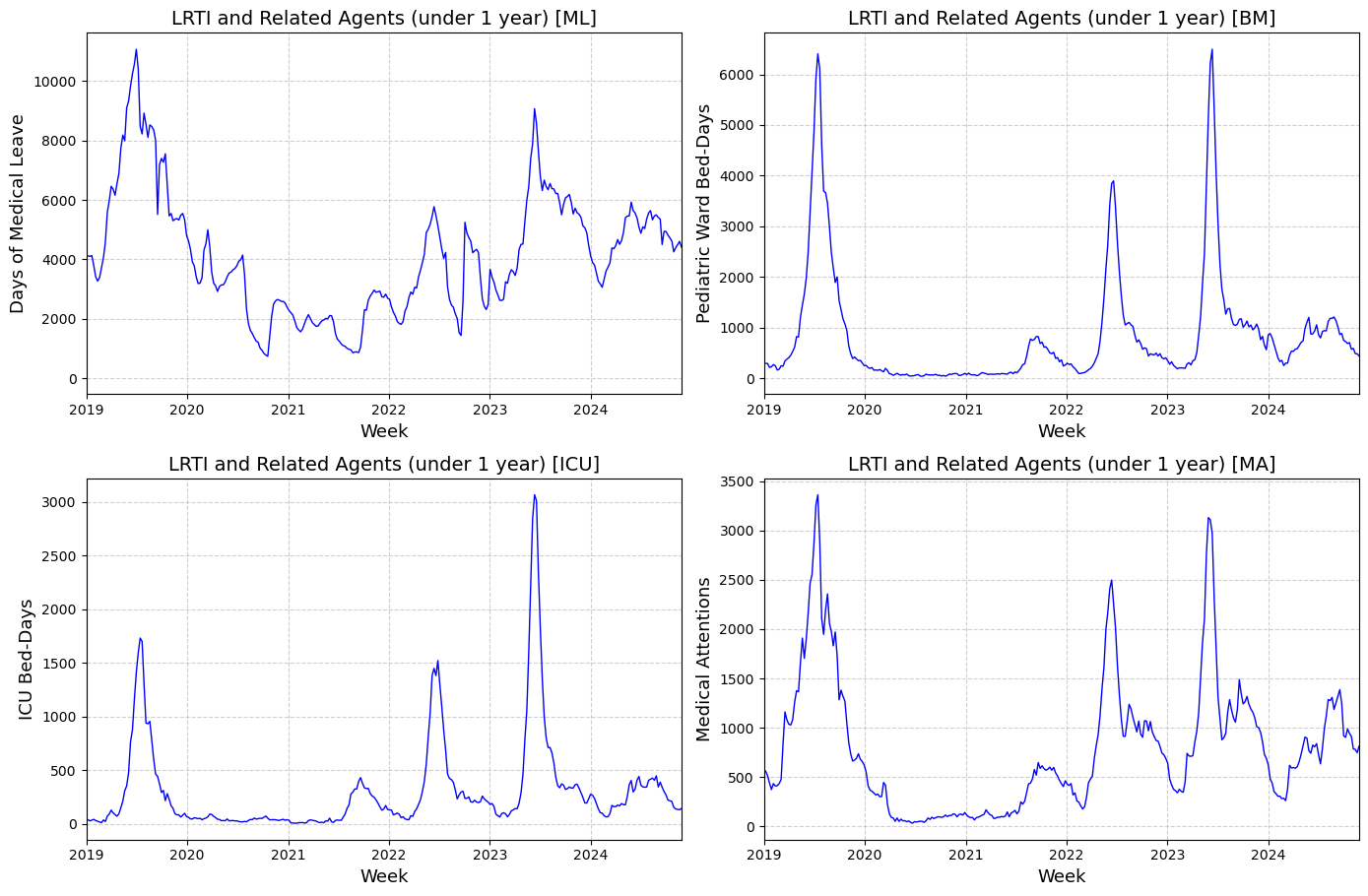


ML: medical leave; MA: medical attention; BM: basic and intermediate bed-day; ICU: intensive care unit.

**Figure S1.** Weekly time series of outcomes.


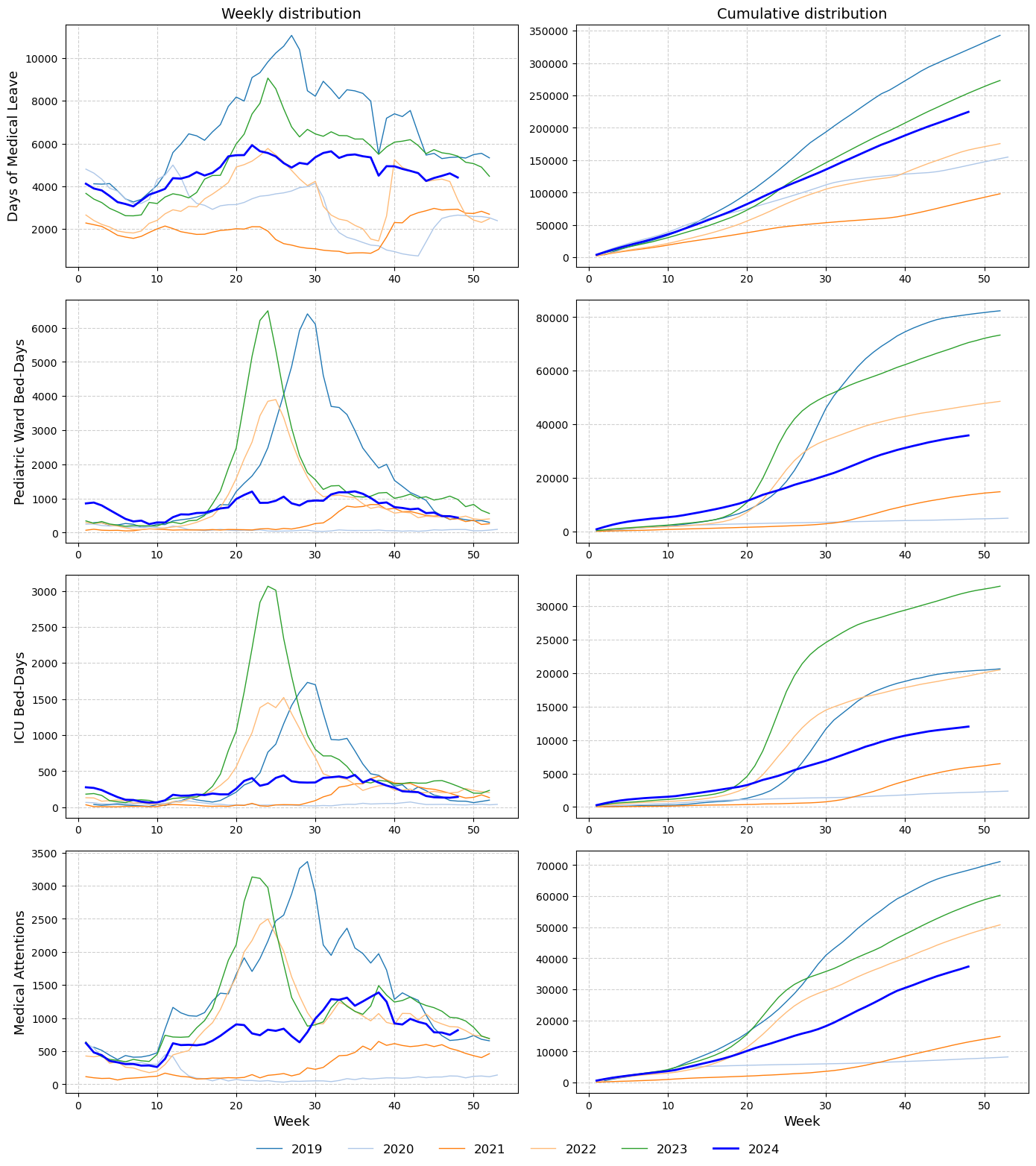


ML: medical leave; MA: medical attention; BM: basic and intermediate bed-day; ICU: intensive care unit.

**Figure S2.** Weekly and Cumulative Distribution of Treated LRTI and Related Agents Units by year.

The first column shows the weekly distribution and the second one the cumulative distribution.

Rows correspond to outcomes: ML (first row), BM (second row), ICU (third row), and MA (fourth row).

| **Year** | | **2019** | **2020** | **2021** | **2022** | **2023** | **2024** |
| --- | --- | --- | --- | --- | --- | --- | --- |
| **ML** | | | | | | | |
| Number of Medical Leaves | | 2 106·92 | 819·63 | 612·16 | 1 049·16 | 1686·82 | 1467·49 |
| Mean Number of Leave Days (std) | | 8·14 (4·65) | 9·92 (6·29) | 9·40 (5·66) | 9·26 (6·05) | 9·68 (6·66) | 10·19 (7·29) |
| **MA** | | | | | | | |
| Number of Medical Attentions | | 3 410·47 | 422·71 | 836·00 | 2 682·10 | 3462·29 | 2 411·79 |
| **BM** | | | | | | | |
| Admissions | | 995·30 | 58·72 | 242·79 | 691·48 | 1125·14 | 706·52 |
| Mean Length of Stay (std) | | 4·82 (8·94) | 5·63 (21·88) | 4·49 (6·15) | 4·58 (5·31) | 4·72 (6·17) | 4·26 (5·31) |
| Cohort | | | | | | | |
| Catch-up | Admissions | 452·55 | 25·39 | 60·30 | 303·16 | 526·23 | 313·87 |
|  | Mean Length of Stay (std) | 4·71 (5·59) | 6·09 (18·48) | 4·38 (5·76) | 4·55 (4·62) | 4·76 (6·84) | 4·40 (5·70) |
| Seasonal | Admissions | 382·42 | 11·64 | 148·30 | 279·97 | 386·92 | 184·27 |
|  | Mean Length of Stay (std) | 4·96 (12·21) | 4·60 (5·46) | 4·44 (4·06) | 4·53 (4·80) | 4·61 (5·06) | 4·22 (5·67) |
| Others | Admissions | 160·33 | 21·69 | 34·18 | 108·34 | 211·99 | 208·36 |
|  | Mean Length of Stay (std) | 4·75 (7·20) | 5·63 (29·68) | 4·91 (11·76) | 4·78 (7·79) | 4·82 (6·23) | 4·09 (4·1) |
| **ICU** | | | | | | | |
| Admissions | | 209·24 | 12·57 | 71·75 | 216·32 | 400·25 | 173·26 |
| Mean Length of Stay (std) | | 7·68 (16·35) | 11·58 (42·84) | 8·17 (23·26) | 8·73 (17·82) | 8·35 (15·46) | 7·88 (16·76) |
| Cohort | | | | | | | |
| Catch-up | Admissions | 83·16 | 5·33 | 13·93 | 83·09 | 175·85 | 73·88 |
|  | Mean Length of Stay (std) | 8·17 (19·36) | 8·50 (15·95) | 12·21 (40·23) | 8·78 (18·74) | 8·02 (14·24) | 8·40 (17·56) |
| Seasonal | Admissions | 99·01 | 3·64 | 47·55 | 103·70 | 157·81 | 51·64 |
|  | Mean Length of Stay (std) | 7·70 (15·54) | 5·80 (5·27) | 7·02 (10·68) | 8·72 (16·20) | 7·58 (10·32) | 5·72 (6·57) |
| Others | Admissions | 27·07 | 3·59 | 10·27 | 29·53 | 67·16 | 47·73 |
|  | Mean Length of Stay (std) | 6·13 (5·36) | 22·00 (76·96) | 8·03 (32·25) | 8·62 (20·40) | 11·04 (25·22) | 9·42 (22·12) |

L: medical leave; MA: medical attention; BM: basic and intermediate bed-day; ICU: intensive care unit.

**Table S2.** Summary of characteristics of each outcome across study period, per 10 000 births.

**Augmented Synthetic Control Method (ASCM)**

Notation: For [
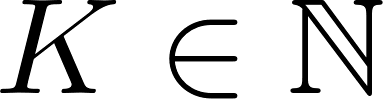
](https://www.codecogs.com/eqnedit.php?latex=K%5Cin%5Cmathbb%7BN%7D#0), we notate [
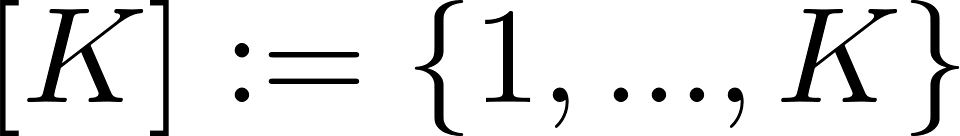
](https://www.codecogs.com/eqnedit.php?latex=%5BK%5D%3A%3D%5C%7B1%2C...%2CK%5C%7D#0).

Problem Definition

We study the problem of estimating the effect of an intervention on a single treated unit, observed over multiple time periods.

Let [
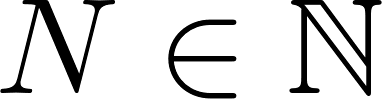
](https://www.codecogs.com/eqnedit.php?latex=N%20%5Cin%20%5Cmathbb%7BN%7D#0), [
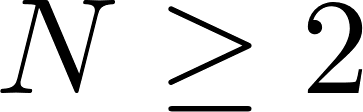
](https://www.codecogs.com/eqnedit.php?latex=N%20%5Cgeq%202#0), denote the number of units in the population. Let [
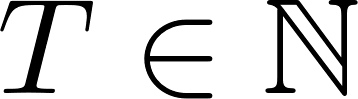
](https://www.codecogs.com/eqnedit.php?latex=T%20%5Cin%20%5Cmathbb%7BN%7D#0), [
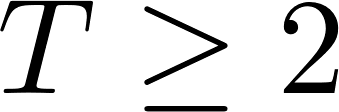
](https://www.codecogs.com/eqnedit.php?latex=T%20%5Cgeq%202#0), denote the number of time periods, and let [
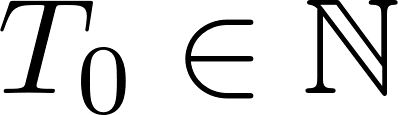
](https://www.codecogs.com/eqnedit.php?latex=T_0%20%5Cin%20%5Cmathbb%7BN%7D#0), with [
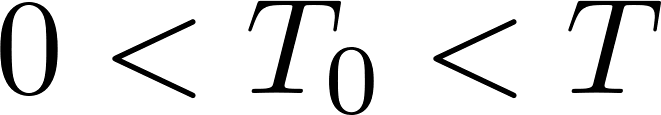
](https://www.codecogs.com/eqnedit.php?latex=0%20%3C%20T_0%20%3C%20T#0), denote the number of pre-intervention periods. Define [
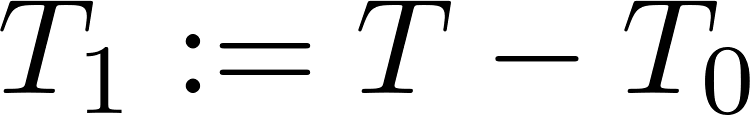
](https://www.codecogs.com/eqnedit.php?latex=T_1%20%3A%3D%20T%20-%20T_0#0), the number of post-intervention periods.

Let [
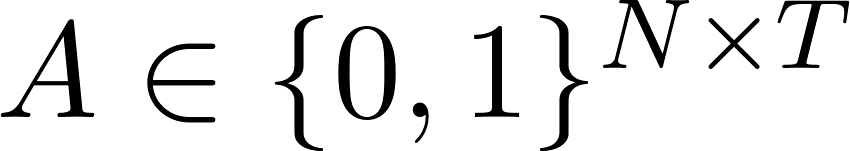
](https://www.codecogs.com/eqnedit.php?latex=A%20%5Cin%20%5C%7B0%2C1%5C%7D%5E%7BN%20%5Ctimes%20T%7D#0) be the treatment assignment matrix, where [
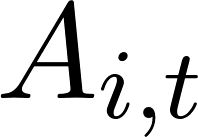
](https://www.codecogs.com/eqnedit.php?latex=A_%7Bi%2Ct%7D#0) is the treatment status of unit [
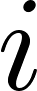
](https://www.codecogs.com/eqnedit.php?latex=i#0) at time [
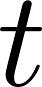
](https://www.codecogs.com/eqnedit.php?latex=t#0), with [
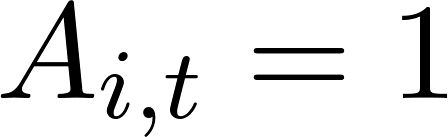
](https://www.codecogs.com/eqnedit.php?latex=A_%7Bi%2Ct%7D%3D1#0) if treated and [
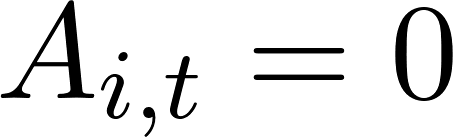
](https://www.codecogs.com/eqnedit.php?latex=A_%7Bi%2Ct%7D%3D0#0) otherwise, for [
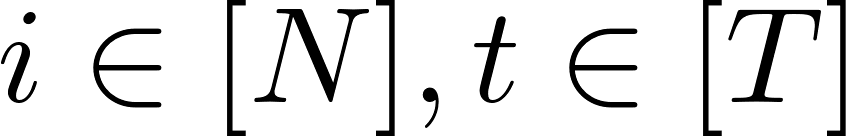
](https://www.codecogs.com/eqnedit.php?latex=i%20%5Cin%20%5BN%5D%2C%20t%20%5Cin%20%5BT%5D#0).

Let [
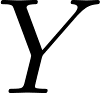
](https://www.codecogs.com/eqnedit.php?latex=Y#0) be a random matrix of dimension [
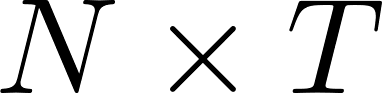
](https://www.codecogs.com/eqnedit.php?latex=N%20%5Ctimes%20T#0), where [
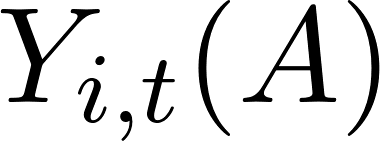
](https://www.codecogs.com/eqnedit.php?latex=Y_%7Bi%2Ct%7D(A)#0) denotes the potential outcome of unit [
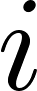
](https://www.codecogs.com/eqnedit.php?latex=i#0) at time [
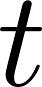
](https://www.codecogs.com/eqnedit.php?latex=t#0) under treatment status [
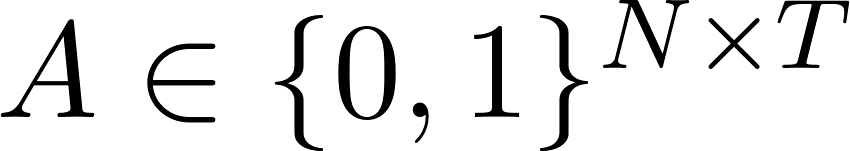
](https://www.codecogs.com/eqnedit.php?latex=A%20%5Cin%20%5C%7B0%2C1%5C%7D%5E%7BN%20%5Ctimes%20T%7D#0).

We assume that treatment assignment is independent of potential outcomes:

[
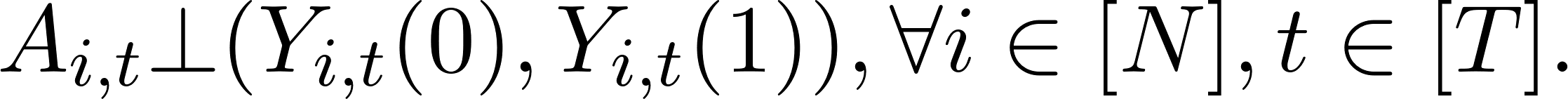
](https://www.codecogs.com/eqnedit.php?latex=A_%7Bi%2Ct%7D%5Cbot%20(Y_%7Bi%2Ct%7D(0)%2CY_%7Bi%2Ct%7D(1))%2C%20%5Cforall%20i%20%5Cin%20%5BN%5D%2C%20t%20%5Cin%20%5BT%5D.#0)

Furthermore, we assume that:

[
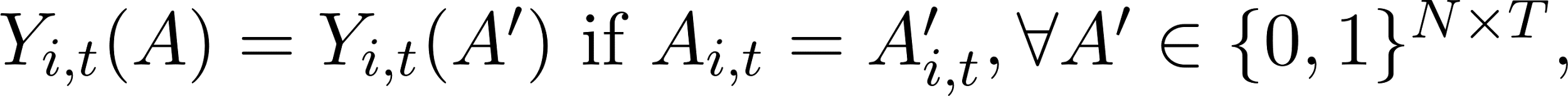
](https://www.codecogs.com/eqnedit.php?latex=Y_%7Bi%2Ct%7D(A)%3DY_%7Bi%2Ct%7D(A')%20%5Ctext%7B%20if%20%7D%20A_%7Bi%2Ct%7D%3DA'_%7Bi%2Ct%7D%2C%20%5Cforall%20A'%5Cin%5C%7B0%2C1%5C%7D%5E%7BN%5Ctimes%20T%7D%2C#0) (1)

i.e., the potential outcome of each unit depends only on its own treatment status, not on that of other units (no interference).

Let [
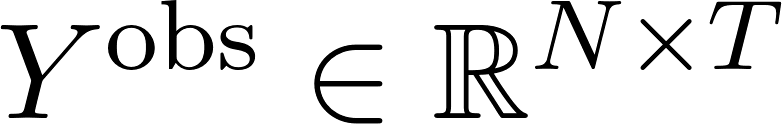
](https://www.codecogs.com/eqnedit.php?latex=Y%5E%7B%5Ctext%7Bobs%7D%7D%20%5Cin%20%5Cmathbb%7BR%7D%5E%7BN%20%5Ctimes%20T%7D#0) denote the observed outcome matrix. We further assume consistency:

[
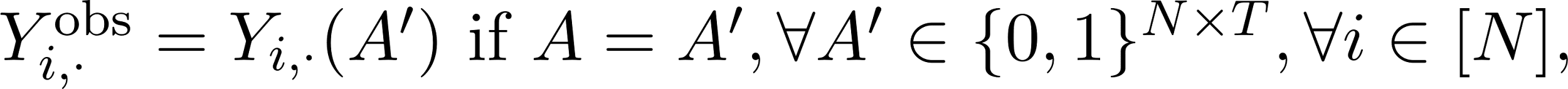
](https://www.codecogs.com/eqnedit.php?latex=Y_%7Bi%2C%5Ccdot%7D%5E%7B%5Ctext%7Bobs%7D%7D%3DY_%7Bi%2C%5Ccdot%7D(A')%20%5Ctext%7B%20if%20%7D%20A%3DA'%2C%20%5Cforall%20A'%5Cin%5C%7B0%2C1%5C%7D%5E%7BN%5Ctimes%20T%7D%2C%20%5Cforall%20i%5Cin%5BN%5D%2C#0) (2)

i.e., the potential outcome given by the treated status is exactly the observed one.

Together, assumptions (1) and (2) correspond to the Stable Unit Treatment Value Assumption (SUTVA) [s1], which rules out spillover effects.

In this study, treatment assignment was almost surely, i.e., [
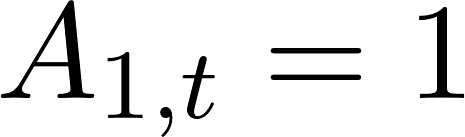
](https://www.codecogs.com/eqnedit.php?latex=A_%7B1%2Ct%7D%3D1#0) if [
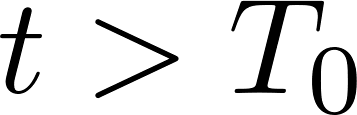
](https://www.codecogs.com/eqnedit.php?latex=t%3ET_0#0) and [
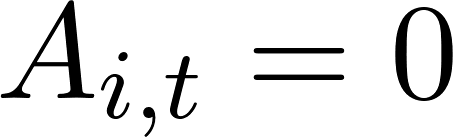
](https://www.codecogs.com/eqnedit.php?latex=A_%7Bi%2Ct%7D%3D0#0) if [
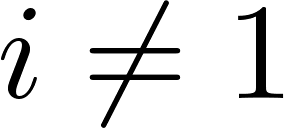
](https://www.codecogs.com/eqnedit.php?latex=i%5Cneq%201#0) or [
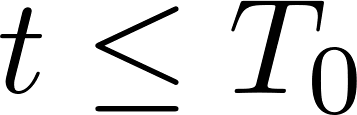
](https://www.codecogs.com/eqnedit.php?latex=t%5Cleq%20T_0#0).

That is, only unit [
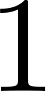
](https://www.codecogs.com/eqnedit.php?latex=1#0) was treated, and only after time [
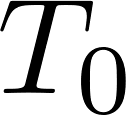
](https://www.codecogs.com/eqnedit.php?latex=T_0#0). Following [s2] and [16], unit [
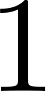
](https://www.codecogs.com/eqnedit.php?latex=1#0) is referred to as the treated unit, while the remaining units form the donor pool.

Let [
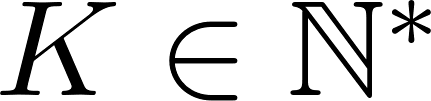
](https://www.codecogs.com/eqnedit.php?latex=K%20%5Cin%20%5Cmathbb%7BN%7D%5E*#0) denotes the number of observed covariates per unit, unaffected by the intervention. Let [
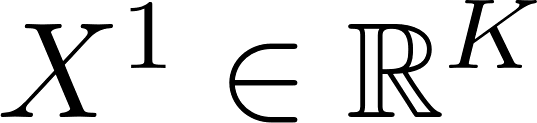
](https://www.codecogs.com/eqnedit.php?latex=X%5E1%20%5Cin%20%5Cmathbb%7BR%7D%5EK#0) be the vector of covariates for the treated unit, and [
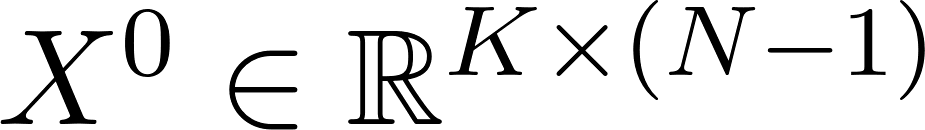
](https://www.codecogs.com/eqnedit.php?latex=X%5E0%20%5Cin%20%5Cmathbb%7BR%7D%5E%7BK%20%5Ctimes%20(N-1)%7D#0) the covariate matrix for the donor pool, where [
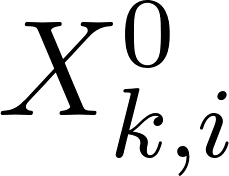
](https://www.codecogs.com/eqnedit.php?latex=X%5E0_%7Bk%2Ci%7D#0) is the value of covariate [
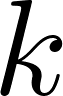
](https://www.codecogs.com/eqnedit.php?latex=k#0) for donor unit [
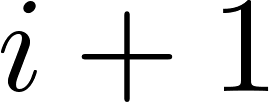
](https://www.codecogs.com/eqnedit.php?latex=i%2B1#0), with [
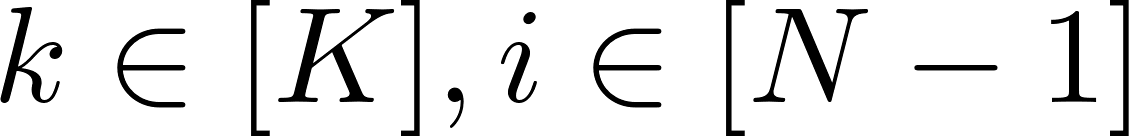
](https://www.codecogs.com/eqnedit.php?latex=k%20%5Cin%20%5BK%5D%2C%20i%20%5Cin%20%5BN-1%5D#0).

For [
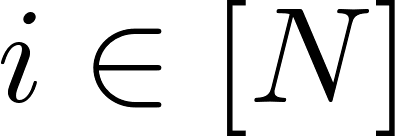
](https://www.codecogs.com/eqnedit.php?latex=i%20%5Cin%20%5BN%5D#0) and [
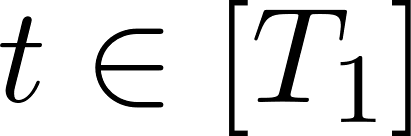
](https://www.codecogs.com/eqnedit.php?latex=t%20%5Cin%20%5BT_1%5D#0), the individual treatment effect at time [
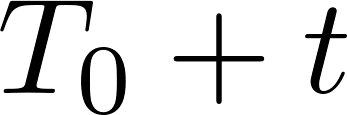
](https://www.codecogs.com/eqnedit.php?latex=T_0%2Bt#0) is defined as:

[
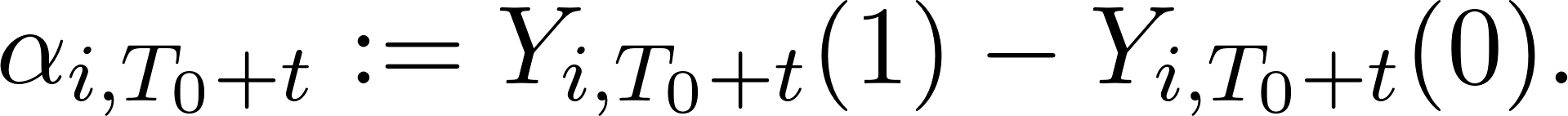
](https://www.codecogs.com/eqnedit.php?latex=%5Calpha_%7Bi%2CT_0%2Bt%7D%20%3A%3D%20Y_%7Bi%2CT_0%2Bt%7D(1)-Y_%7Bi%2CT_0%2Bt%7D(0).#0)

The objective is to estimate the treatment effect path of the treated unit

[
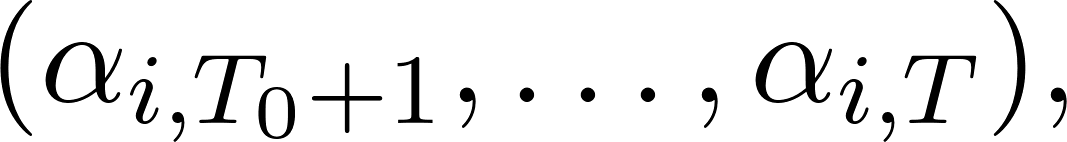
](https://www.codecogs.com/eqnedit.php?latex=(%5Calpha_%7Bi%2CT_0%2B1%7D%2C%5Cdots%2C%5Calpha_%7Bi%2CT%7D)%2C#0)

where

[
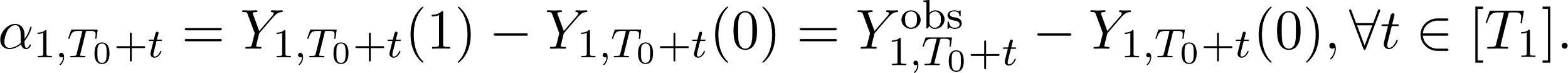
](https://www.codecogs.com/eqnedit.php?latex=%5Calpha_%7B1%2CT_0%2Bt%7D%20%3D%20Y_%7B1%2CT_0%2Bt%7D(1)-Y_%7B1%2CT_0%2Bt%7D(0)%3DY_%7B1%2CT_0%2Bt%7D%5E%7B%5Ctext%7Bobs%7D%7D-Y_%7B1%2CT_0%2Bt%7D(0)%2C%5Cforall%20t%5Cin%5BT_1%5D.#0)

Thus, the central problem reduces to estimating the unobserved counterfactual series [
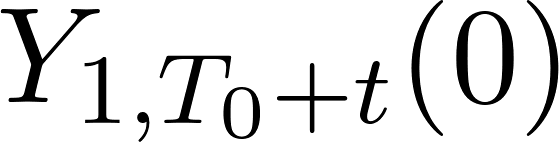
](https://www.codecogs.com/eqnedit.php?latex=Y_%7B1%2CT_0%2Bt%7D(0)#0) for [
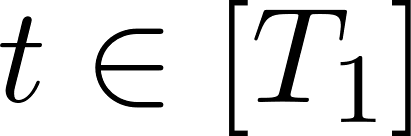
](https://www.codecogs.com/eqnedit.php?latex=t%20%5Cin%20%5BT_1%5D#0).

Synthetic Control Method (SCM)

The Synthetic Control Method (SCM) [s2,16] approximates the untreated outcome of the treated unit by a convex combination of the control units. It is assumed that exists a vector of weights

[
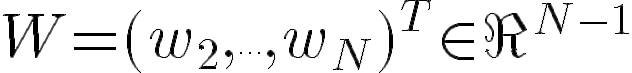
](http://www.texrendr.com/?eqn=W%3D(w_2%2C%5Cdots%2Cw_%7BN%7D)%5ET%5Cin%5CR%5E%7BN-1%7D#0), with [
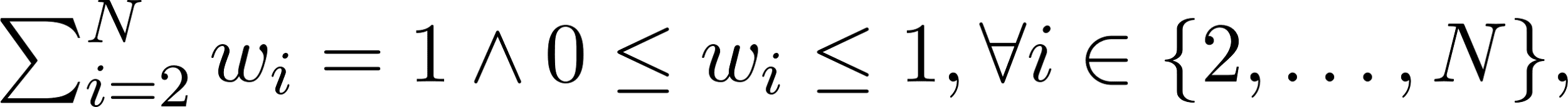
](https://www.codecogs.com/eqnedit.php?latex=%5Csum_%7Bi%3D2%7D%5E%7BN%7Dw_i%3D1%20%5Cwedge%200%5Cleq%20w_i%5Cleq%201%2C%5Cforall%20i%5Cin%5C%7B2%2C%5Cdots%2CN%5C%7D%2C#0)

such that

[
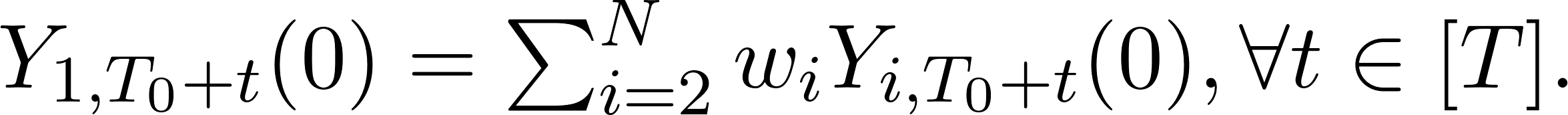
](https://www.codecogs.com/eqnedit.php?latex=Y_%7B1%2CT_0%2Bt%7D(0)%20%3D%20%5Csum_%7Bi%3D2%7D%5EN%20w_i%20Y_%7Bi%2CT_0%2Bt%7D(0)%2C%20%5Cforall%20t%5Cin%20%5BT%5D.#0)

The vector [
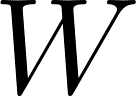
](https://www.codecogs.com/eqnedit.php?latex=W#0) is called synthetic control.

To estimate the synthetics series, then we estimate the synthetic control by minimizing the discrepancy between the treaded unit and the synthetic series in terms of the pre-treatment values and covariates. This is for [
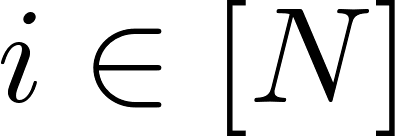
](https://www.codecogs.com/eqnedit.php?latex=i%5Cin%5BN%5D#0), let [
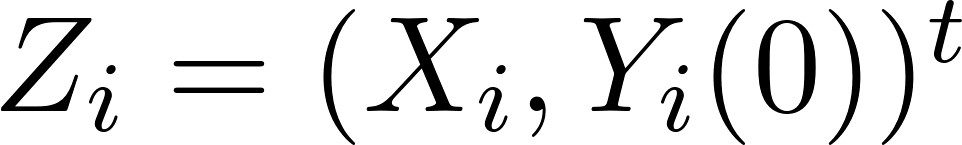
](https://www.codecogs.com/eqnedit.php?latex=Z_i%3D(X_i%2CY_%7Bi%7D(0))%5Et#0) the vector of dimension [
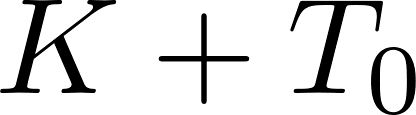
](https://www.codecogs.com/eqnedit.php?latex=K%2BT_0#0) containing the covariables and pre-intervention outputs of unit [
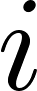
](https://www.codecogs.com/eqnedit.php?latex=i#0). Let [
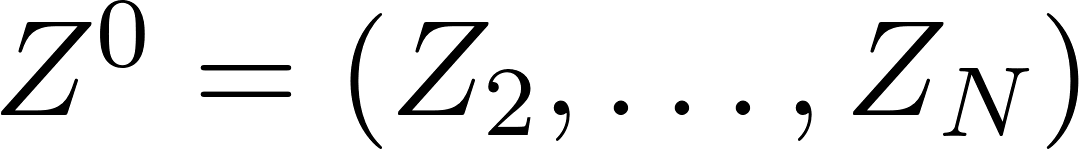
](https://www.codecogs.com/eqnedit.php?latex=Z%5E0%3D(Z_2%2C%5Cdots%2CZ_N)#0) the matrix of dimension [
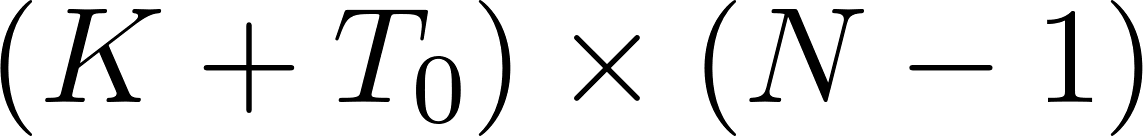
](https://www.codecogs.com/eqnedit.php?latex=(K%2BT_0)%5Ctimes%20(N-1)#0) containing the covariables and pre-intervention outputs of the control units. Then, [
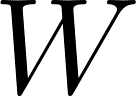
](https://www.codecogs.com/eqnedit.php?latex=W#0) is estimated as

[
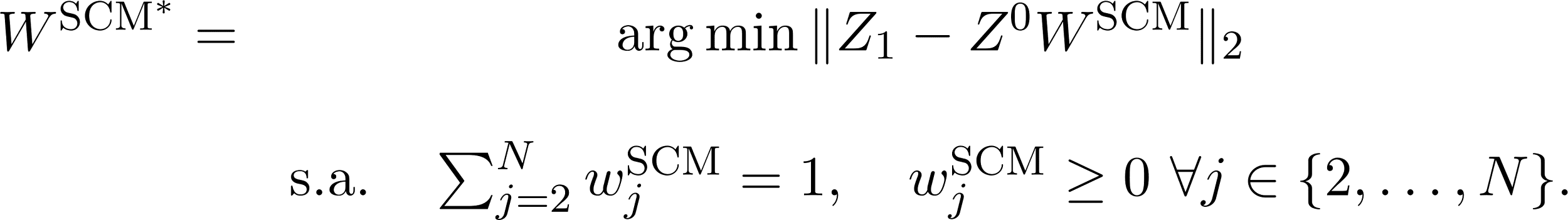
](https://www.codecogs.com/eqnedit.php?latex=%5Cbegin%7Bmatrix%7D%20W%5E%7B%5Ctext%7BSCM%7D%5E*%7D%20%3D%26%20%5Carg%5Cmin%20%5C%7CZ_1%20-%20Z%5E0%20W%5E%7B%5Ctext%7BSCM%7D%7D%5C%7C_2%20%5C%5C%5C%5C%20%26%20%5C%5C%5C%5C%20%26%20%5Ctext%7Bs.a.%7D%20%5Cquad%20%20%5Csum_%7Bj%3D2%7D%5E%7BN%7D%20w%5E%7B%5Ctext%7BSCM%7D%7D_j%20%3D%201%2C%5Cquad%20w%5E%7B%5Ctext%7BSCM%7D%7D_j%20%5Cgeq%200%20%5C%20%5Cforall%20j%5Cin%5C%7B2%2C%5Cdots%2CN%5C%7D.%20%5Cend%7Bmatrix%7D#0)

Then, the SCM estimate of the treatment effect in period [
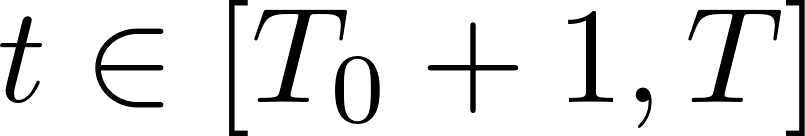
](https://www.codecogs.com/eqnedit.php?latex=t%5Cin%5BT_0%2B1%2CT%5D#0) is

[
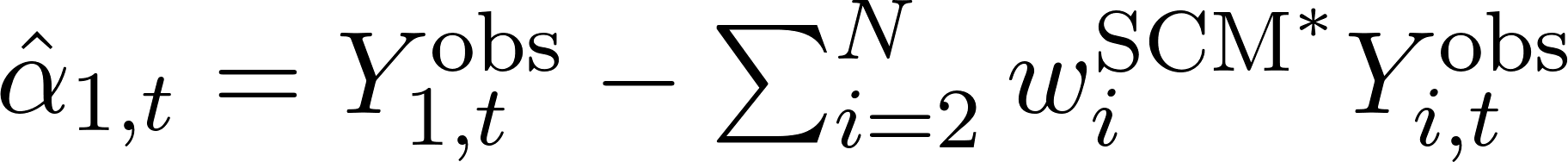
](https://www.codecogs.com/eqnedit.php?latex=%20%5Chat%7B%5Calpha%7D_%7B1%2Ct%7D%20%3D%20Y_%7B1%2Ct%7D%5E%7B%5Ctext%7Bobs%7D%7D-%5Csum%5EN_%7Bi%3D2%7D%20w%5E%7B%5Ctext%7BSCM%7D%5E*%7D_i%20Y_%7Bi%2Ct%7D%5E%7B%5Ctext%7Bobs%7D%7D#0)

While SCM performs well when pre-treatment fit is good, it may produce biased estimates if no convex combination of donors closely matches the treated unit.

Augmented Synthetic Control Method (ASCM)

The Augmented Synthetic Control Method (ASCM) [14] extends SCM by correcting for imperfect pre-treatment fit using a regression adjustment. Specifically, after computing the SCM weights, ASCM augments the estimate with a bias-correction term derived from a penalized regression of post-treatment outcomes on pre-treatment outcomes and covariates. This is, the augmented estimated is

[
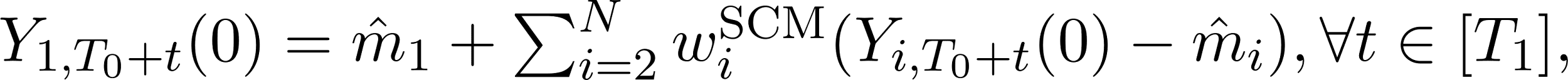
](https://www.codecogs.com/eqnedit.php?latex=Y_%7B1%2CT_0%2Bt%7D(0)%20%3D%20%5Chat%7Bm%7D_%7B1%7D%20%2B%20%5Csum_%7Bi%3D2%7D%5EN%20w%5E%7B%5Ctext%7BSCM%7D%7D_i%20(Y_%7Bi%2CT_0%2Bt%7D(0)-%5Chat%7Bm%7D_%7Bi%7D)%2C%20%5Cforall%20t%5Cin%20%5BT_1%5D%2C#0)

where [
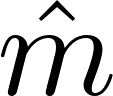
](https://www.codecogs.com/eqnedit.php?latex=%5Chat%7Bm%7D#0) is a post-treatment control potential outcome. In this study we used a control that is linear for pre-treatments outcomes, i.e., [
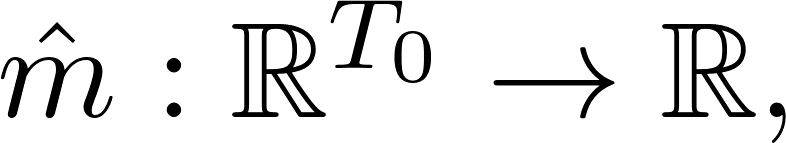
](https://www.codecogs.com/eqnedit.php?latex=%5Chat%7Bm%7D%3A%5Cmathbb%7BR%7D%5E%7BT_0%7D%5Cto%5Cmathbb%7BR%7D%2C#0), with

[
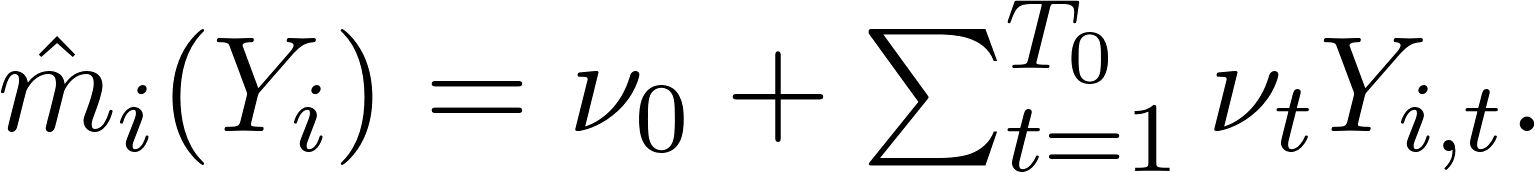
](https://www.codecogs.com/eqnedit.php?latex=%5Chat%7Bm%7D_i(Y_i)%3D%5Cnu_0%20%2B%20%5Csum_%7Bt%3D1%7D%5E%7BT_0%7D%20%5Cnu_t%20Y_%7Bi%2Ct%7D.#0)

Following [14], we estimate the synthetic control with the Ridge ASCM, where the minimization problem penalizes the difference between the SCM and the adjusted weights from the ASCM, i.e.,

[
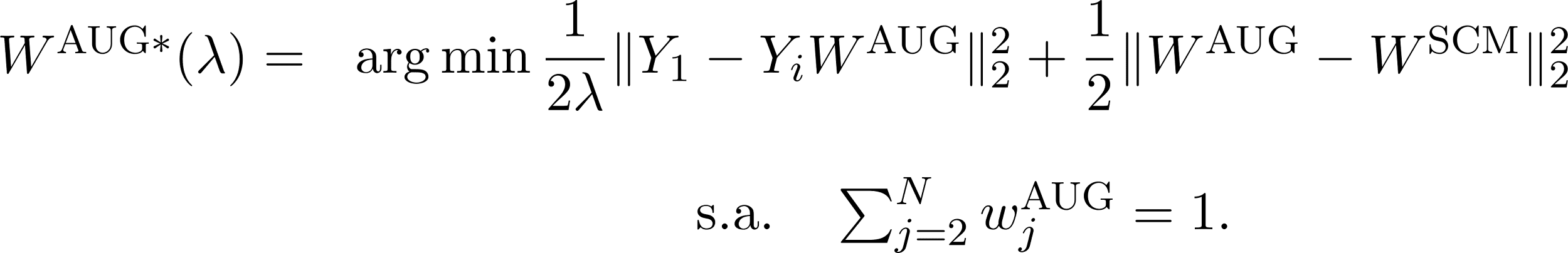
](https://www.codecogs.com/eqnedit.php?latex=%5Cdisplaystyle%7B%5Cbegin%7Bmatrix%7D%20W%5E%7B%5Ctext%7BAUG%7D*%7D(%5Clambda)%20%3D%26%20%5Carg%5Cmin%20%5Cdfrac%7B1%7D%7B2%5Clambda%7D%5C%7CY_1%20-%20Y_i%20W%5E%7B%5Ctext%7BAUG%7D%7D%5C%7C%5E2_2%20%2B%20%5Cdfrac%7B1%7D%7B2%7D%20%5C%7C%20W%5E%7B%5Ctext%7BAUG%7D%7D-W%5E%7B%5Ctext%7BSCM%7D%7D%5C%7C_2%5E2%20%5C%5C%5C%5C%20%26%20%5C%5C%26%20%5Ctext%7Bs.a.%7D%20%5Cquad%20%20%5Csum_%7Bj%3D2%7D%5E%7BN%7D%20w%5E%7B%5Ctext%7BAUG%7D%7D_j%20%3D%201.%20%5Cend%7Bmatrix%7D%7D#0)

When the treated unit is out of the convex hull, the ASCM allows it to assign negative weights to certain units, in order to extrapolate outside. This level of extrapolation will be equilibrated by the imbalance of pre-treatment fit and the selection of [
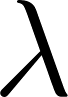
](https://www.codecogs.com/eqnedit.php?latex=%5Clambda#0). This hyperparameter was selected over the grid [
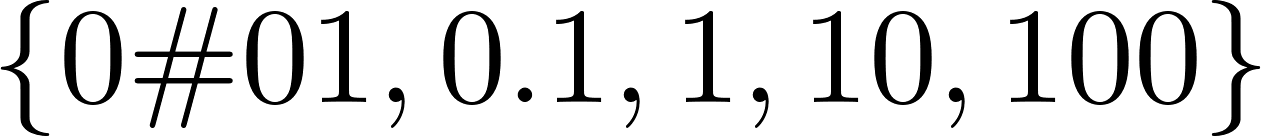
](https://www.codecogs.com/eqnedit.php?latex=%5C%7B0%C2%B701%2C0.1%2C1%2C10%2C100%5C%7D#0) and evaluated in two placebo dates (January 1, 2019, up to the placebo intervention start dates: April 4, 2022, and April 3, 2023). Performance was then assessed over the subsequent 35 weeks (prior to the intervention) by evaluating the trade-off performance of their mean absolute error in the pre-treatment and placebo post-treatment period. For all outcomes was selected [
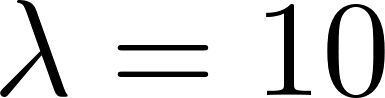
](https://www.codecogs.com/eqnedit.php?latex=%5Clambda%3D10#0).

**Model selection**

To estimate the counterfactual outcomes, we implemented and compared multiple variants of the Synthetic Control Method (SCM): Classic SCM, SCM with Lasso regularization, SCM with Ridge regularization, Penalized SCM, Matching combined with SCM, and Augmented SCM (ASCM). For each specification, dummy control units were added to account for month effects, winter holiday periods, and an intercept.

To evaluate their predictive capacity, the models were trained using data from the pre-treatment period (January 1, 2019, up to the placebo intervention start dates: April 4, 2022, and April 3, 2023). Performance was then assessed over the subsequent 35 weeks, ending on November 28, 2022, and November 27, 2023, respectively. A placebo date refers to an artificially assigned intervention point in a period without treatment, used to test whether the model erroneously detects an effect when none should be present. These placebo intervention dates were selected because they corresponded to RSV seasons without exposure to Nirsevimab, thus providing a setting in which any estimated effects could only reflect model error rather than a true intervention. This procedure allowed a rigorous assessment of whether the models produced valid predictions in the absence of treatment.

Mean absolute errors (MAEs) were calculated for both pre-treatment and post-treatment windows to evaluate performance. The preferred model was selected as the one with the lowest MAE across units. ASCM consistently demonstrated the most stable and reliable performance in these tests and was therefore selected as the final specification for the analysis (see Table S3).

| **Outcome** | **Model** | **Pre-treatment MAE** | **Post-treatment MAE** |
| --- | --- | --- | --- |
| ML | SCM | 1495·23 | 1596·2 |
|  | SCM with Lasso regularization | 1523·77 | 1584·41 |
|  | SCM with Ridge regularization | 1523·77 | 1584·41 |
|  | Penalized SCM | 1523·77 | 1584·41 |
|  | Matching combined with SCM | 2701·27 | 3930·38 |
|  | ASCM | 261·78 | 859·4 |
| MA | SCM | 534·93 | 418·66 |
|  | SCM with Lasso regularization | 536·61 | 417·84 |
|  | SCM with Ridge regularization | 536·61 | 417·84 |
|  | Penalized SCM | 536·61 | 417·84 |
|  | Matching combined with SCM | 533·27 | 1218·82 |
|  | ASCM | 76·46 | 262·1 |
| BM | SCM | 1126·15 | 1008·54 |
|  | SCM with Lasso regularization | 1269·53 | 1224·14 |
|  | SCM with Ridge regularization | 1269·53 | 1224·14 |
|  | Penalized SCM | 1269·53 | 1224·14 |
|  | Matching combined with SCM | 541·21 | 1379·25 |
|  | ASCM | 168·12 | 676·91 |
|  | SCM | 603·35 | 613·52 |
|  | SCM with Lasso regularization | 577·79 | 618·93 |
|  | SCM with Ridge regularization | 577·79 | 618·93 |
|  | Penalized SCM | 577·79 | 618·93 |
|  | Matching combined with SCM | 163·73 | 614·77 |
|  | ASCM | 56·43 | 339·13 |

**Table S3.** Pre-treatment and post-treatment mean absolute error in test dates (mean of both years).

**Control Units**

Control units from inpatient care and days of medical leave were defined by combinations of age groups and diagnostic categories, following the framework of [15], with specific adaptations (see Table S4). Additional control series were drawn from outpatient care and viral surveillance data, as follows:

- Outpatient care: A00–A09 (intestinal infectious diseases), J00–J06 and J40–J46 (non-RSV respiratory diseases), and I00–I99 (circulatory system diseases).
- Surveillance: Weekly virus-positive counts from ILI and SARI reports, including RSV, adenovirus, metapneumovirus, influenza A/B, parainfluenza, SARS-CoV-2, and rhinovirus.

Age group definitions were harmonized based on the outpatient structure, with exceptions for ILI/SARI reporting constraints:

- Medical leaves: <1 year.
- Surveillance: all-ages for ILI/SARI overall; for influenza: 0–2, 2–4, 5–19, 20–39, 40–59, 60+ years.
- Medical attentions: <1 year, 5–14, 15–64, and 65+ years.
- Inpatient care: <1 year, 5–14, 15–64, and 65+ years.

Perinatal respiratory and cardiac diagnoses (ICD-10 P20–P29) and LRTI and related agents outcomes in children aged 1–4 years were excluded from the analysis to avoid potential spillover effects.

| **ICD-10 Codes** | **Description** |
| --- | --- |
| **Chapter** | |
| P00–P96 (Excluding P20–P29) | Certain conditions originating in the perinatal period |
| C00–D48 | Neoplasms |
| D50–D89 | Diseases of the blood and blood-forming organs and immune disorders |
| E00–E90 | Endocrine, nutritional, and metabolic diseases |
| F00–F99 | Mental and behavioral disorders |
| G00–G99 | Diseases of the nervous system |
| I00–I99 | Diseases of the circulatory system |
| K00–K93 | Diseases of the digestive system |
| L00–L99 | Diseases of the skin and subcutaneous tissue |
| M00–M99 | Musculoskeletal and connective tissue diseases |
| N00–N99 | Diseases of the genitourinary system |
| Q00–Q99 | Congenital malformations and chromosomal abnormalities |
| R00–R99 | Symptoms and abnormal findings not elsewhere classified |
| S00–T98 | Injury, poisoning, and other consequences of external causes |
| U00–U99 | Codes for special purposes |
| V01–Y98 | External causes of morbidity and mortality |
| Z00–Z99 | Factors influencing health status and contact with health services |
| H00–H95 | Eye and ear diseases |
| **Group** | |
| A00–A09 | Intestinal infectious diseases |
| B20–B24 | HIV disease |
| E10–E14 | Diabetes mellitus |
| E40–E46 | Malnutrition |
| I60–I69 | Cerebrovascular diseases |
| P05–P08 | Disorders of newborn related to gestation and growth |
| J00–J06,J30–J39,J40–J46 | RSV-non-related respiratory diseases |
| **Category** | |
| A17 | Tuberculosis of the nervous system |
| A18 | Tuberculosis of other organs |
| A19 | Miliary tuberculosis |
| K35 | Acute appendicitis |
| K80 | Cholelithiasis (gallstones) |
| N39 | Other disorders of the urinary system |

**Table S4.** ICD-10 codes aggrupation used to create the control units.

**Covariates**

To improve the characterization of each unit, and thereby the accuracy of counterfactual estimates, a broad set of covariates was constructed using information from medical leave, inpatient, and outpatient records, selected according to both temporal and group-specific relevance. Covariates refer to auxiliary variables that are not the main outcomes of interest, but are related to them and help explain their variation. In the context of synthetic control methods, covariates play a crucial role by providing additional information on the characteristics of the treated and control units prior to the intervention. By aligning units not only on their pre-intervention outcomes but also on these related variables, covariates improve the plausibility that the synthetic control reproduces both observed and unobserved drivers of the treated unit’s trajectory.

Given the absence of a standardized framework for covariate selection in synthetic control methods [s3], we adopted a data-driven approach to identify the most relevant covariates for each treated unit. This strategy ensured that the selected covariates captured patterns closely associated with the treated outcomes while avoiding the inclusion of irrelevant or weak predictors. All candidate variables were tested for their predictive capacity, and those contributing most to explaining the outcome were retained.

Covariates Definition

*Temporal Covariates*

These variables captured both the long-term evolution of outcomes (trends) and their seasonal patterns in the treated and control units. They were calculated by aggregating the main outcomes over different time intervals, i.e., linear transformations of the outcomes:

- Monthly accumulated values: total observed per calendar month.
- Quarterly averages: weekly and monthly averages within each calendar quarter.
- Four-month averages: weekly and monthly averages within each four-month period.
- Quarterly totals: sum of observations per quarter.
- Four-month totals: sum of observations per four-month period.
- Yearly totals: sum of observations per year.
- Yearly averages: monthly averages within each year.
- Calendar features: categorical indicators (month, week, quarter, four-month period) and harmonic features derived from sine and cosine transformations of week and month values. These features ensured that seasonal patterns were captured for each treated unit by transforming week and month with [
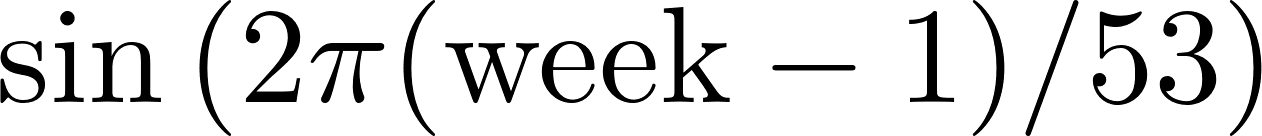
](https://www.codecogs.com/eqnedit.php?latex=%5Csin%5Cleft(2%5Cpi%20(%5Ctext%7Bweek%7D%20-%201)%2F53%5Cright)#0), [
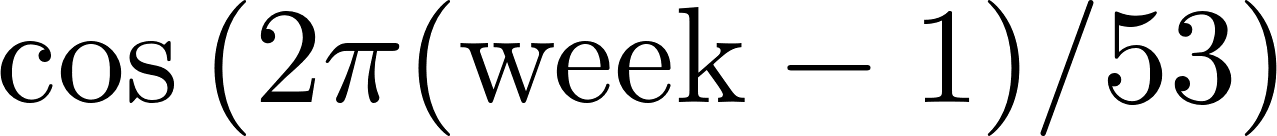
](https://www.codecogs.com/eqnedit.php?latex=%5Ccos%5Cleft(2%5Cpi%20(%5Ctext%7Bweek%7D%20-%201)%2F53%5Cright)#0), [
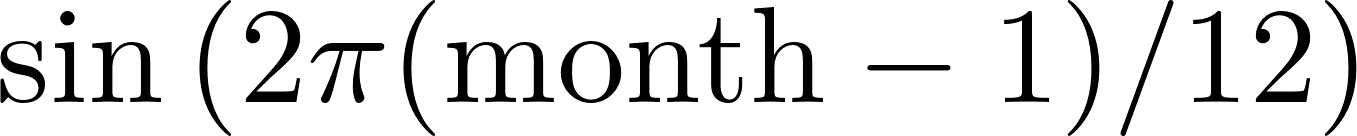
](https://www.codecogs.com/eqnedit.php?latex=%5Csin%5Cleft(2%5Cpi%20(%5Ctext%7Bmonth%7D%20-%201)%2F12%5Cright)#0), and [
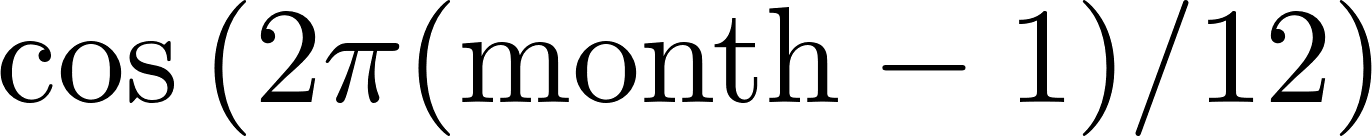
](https://www.codecogs.com/eqnedit.php?latex=%5Ccos%5Cleft(2%5Cpi%20(%5Ctext%7Bmonth%7D%20-%201)%2F12%5Cright)#0).

*Specific Weekly Covariates*

These covariates reflected the geographic distribution of outcomes, capturing spatial heterogeneity across Chile:

- Macro-zone percentage: share of the outcome in each macro-zone.
- Region percentage: share of the outcome in each administrative region.

For medical leave data, geographic characteristics were only available after 2021. To maintain consistency in the pre-treatment period, missing values for earlier years were imputed using average values from inpatient discharges during the same period, restricted to units sharing the same diagnostic group.

*Demographic and Clinical Covariates*

These covariates characterized the demographic composition and clinical severity of each outcome unit on a weekly basis, drawing primarily from inpatient records. To account for unobserved factors in the other time series, these covariates were imputed for each combination of age group and disease category using the corresponding weekly averages from the inpatient dataset. This approach assumed that hospital discharges provided a reliable proxy for the clinical and demographic profile of the broader medical leave population. Variables included:

- Demographic composition: percentage of female patients; percentage of foreign-born patients.
- Clinical indicators: percentage of patients with congenital heart disease (ICD-10 codes considered congenital heart disease: Q200–Q206, Q208–Q209, Q210–Q214, Q218–Q226, Q228–Q234, Q238–Q240, Q242–Q245, Q248–Q260, Q262–Q264.) recorded as a secondary diagnosis.
- Hospitalization characteristics: average number of transfers during hospital stays; average ICU bed-days; average basic/intermediate care bed-days; average length of stay.
- Health insurance affiliation: percentage covered by the public health insurance system (FONASA); percentage covered by private health insurance (ISAPRE).

Covariate Selection Procedure

Because the number of potential covariates was large and their relative importance was uncertain, a data-driven approach was required for selection. The covariate selection procedure was conducted prior to estimation and applied independently to each treated unit, allowing for variation in covariate relevance across outcomes. To ensure comparability across units and stabilize the training process, all features and outcomes were normalized using Min–Max scaling over the pre-intervention period. Although standard SCM approaches typically avoid scaling to preserve interpretability of unit weights [16,17], in this context scaling was necessary due to heterogeneous data sources (medical leave, hospitalizations, outpatient visits) and the wide range of variable magnitudes. While normalization altered the interpretation of estimated weights from absolute contributions to representations of relative temporal dynamics, this trade-off was acceptable given the variability of scales and the need for stable covariate selection. Scaling was performed separately for each treated unit and preserved the temporal ordering of the data.

To evaluate the predictive importance of the covariates, we employed gradient boosting regression models, a family of ensemble learning methods that build predictive functions in a stage-wise manner. These models combine many weak learners—typically shallow decision trees—into a strong learner by fitting each new tree to the residual errors of the previous stage. By iteratively reducing prediction errors, the ensemble gradually minimizes a specified loss function, such as squared error in regression tasks. The advantages of this approach are its flexibility in modeling nonlinear relationships, robustness across heterogeneous data, and high predictive accuracy.

Within this family, the XGBoost algorithm [s4] was selected because of its computational efficiency, scalability, and strong performance in handling complex, nonlinear relationships. Models were trained separately for each treated unit using a time-aware split of the pre-intervention data to ensure that validation respected temporal ordering and avoided look-ahead bias. Embedded cross-validation and early stopping were applied to determine the optimal number of boosting rounds and mitigate overfitting.

Feature importance scores were extracted and applied in a two-stage thresholding process. In the first stage, all variables with importance scores above 1% were retained. In the second stage, the model was retrained using only these variables, and a stricter threshold of 5% was applied to determine the final covariate set.

These thresholds controlled model complexity and reduced dimensionality in the optimization problem while preserving explanatory power.

XGBoost was appropriate in this context because the goal was not to estimate causal effects directly but to identify the most informative covariates for characterizing each treated unit. While the synthetic control method ultimately relied on linear combinations of donor units, the use of a flexible, nonparametric model at the selection stage ensured that important covariates were not omitted simply because of nonlinearities in their predictive contributions. In this way, XGBoost enhanced the robustness of the donor pool construction process and improved comparability between treated and control units.

The resulting covariate sets and their corresponding weights (see Table S5), specific to each treated unit, were then used in the construction of synthetic controls. This procedure was consistent with recent methodological advances in synthetic control and causal inference, which recommend predictive, data-driven covariate selection when theoretical guidance is limited [s5-6,14].

| **Treated Unit** | **Covariate** | **Weight** |
| --- | --- | --- |
| ML | Quarterly Total Days of Medical Leave | 0·78 |
|  | Monthly Total Days of Medical Leave | 0·13 |
| MA | Monthly Total Medical Attentions | 0·55 |
|  | Medical Attentions Rate in Atacama Region | 0·08 |
|  | Medical Attentions Rate in Central Macro-zone | 0·05 |
|  | Four-Month Period Total Medical Attentions | 0·05 |
| BM | Monthly Total Basic/Intermediate Bed Days | 0·53 |
|  | Monthly Total Basic/Intermediate Bed Days (Cosine Transformation) | 0·26 |
|  | Bed Occupancy Rate in Metropolitan Region | 0·06 |
| ICU | Monthly Total ICU Bed Days | 0·72 |
|  | Bed Occupancy Rate in Central Macro-zone | 0·12 |

**Table S5.** Selected covariates and weights for treated units.

**Treatment Effect Statistical Significance**

Following the inference framework of [16, s7], placebo tests in time were applied to the treated unit. Placebo intervention dates were defined between 2022 and 2023, restricted to weeks with at least 35 subsequent weeks of available data. Accordingly, the first placebo test ran from 2022-01-03 to 2022-08-29, and the last from 2023-07-31 to 2024-03-25, yielding a total of 83 placebo tests.

Formally, let [
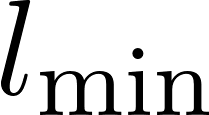
](https://www.codecogs.com/eqnedit.php?latex=l_%7B%5Cmin%7D#0) denote the week index corresponding to 2022-01-03 and [
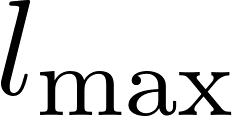
](https://www.codecogs.com/eqnedit.php?latex=l_%7B%5Cmax%7D#0) the week index corresponding to 2023-07-31. Then define

[
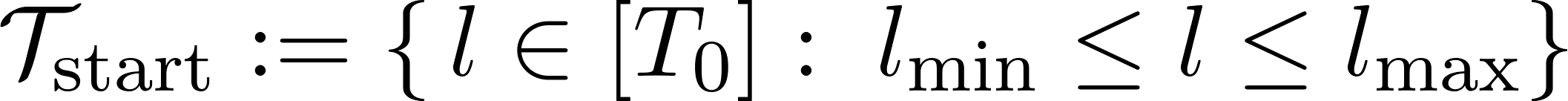
](https://www.codecogs.com/eqnedit.php?latex=%5Cmathcal%7BT%7D_%7B%5Ctext%7Bstart%7D%7D%20%3A%3D%20%5C%7B%20%5C%2C%20l%20%5Cin%20%5BT_0%5D%20%3A%20%5C%3B%20l_%7B%5Cmin%7D%20%5Cleq%20l%20%5Cleq%20l_%7B%5Cmax%7D%20%5C%7D#0)

the set of placebo start dates, where [
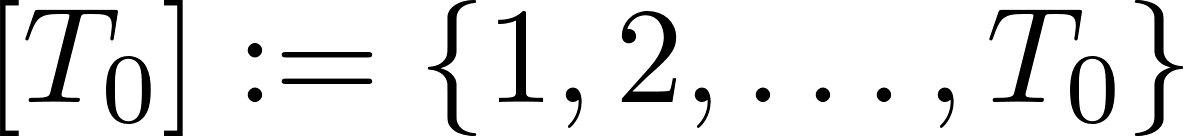
](https://www.codecogs.com/eqnedit.php?latex=%5BT_0%5D%20%3A%3D%20%5C%7B1%2C2%2C%5Cdots%2CT_0%5C%7D#0) denotes the set of all pre-intervention weeks.

For each [
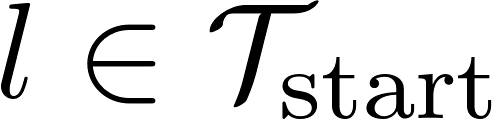
](https://www.codecogs.com/eqnedit.php?latex=l%20%5Cin%20%5Cmathcal%7BT%7D_%7B%5Ctext%7Bstart%7D%7D#0), define the post-treatment evaluation window as
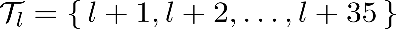
corresponding to the 35 weeks following the placebo intervention.

For each placebo intervention date [
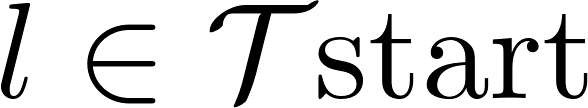
](https://www.codecogs.com/eqnedit.php?latex=l%20%5Cin%20%5Cmathcal%7BT%7D%7B%5Ctext%7Bstart%7D%7D#0), we estimated the treatment effect sequence [
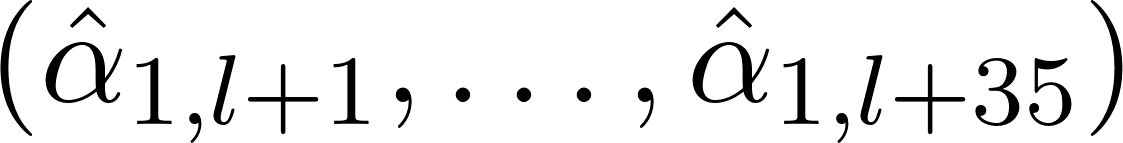
](https://www.codecogs.com/eqnedit.php?latex=(%5Chat%7B%5Calpha%7D_%7B1%2Cl%2B1%7D%2C%20%5Cdots%2C%20%5Chat%7B%5Calpha%7D_%7B1%2Cl%2B35%7D)#0) under the counterfactual assumption that the treated unit had been exposed at time [
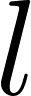
](https://www.codecogs.com/eqnedit.php?latex=l#0).

Under the null hypothesis of no treatment effect,

[
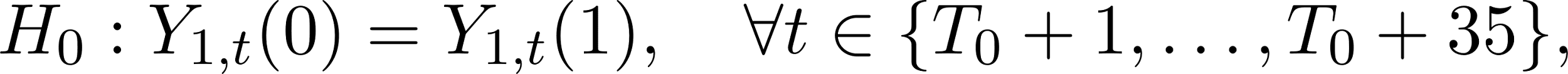
](https://www.codecogs.com/eqnedit.php?latex=H_0%3A%20Y_%7B1%2Ct%7D(0)%20%3D%20Y_%7B1%2Ct%7D(1)%2C%20%5Cquad%20%5Cforall%20t%5Cin%5C%7BT_0%2B1%2C%5Cdots%2CT_0%2B35%5C%7D%2C#0)

the estimated effect at the actual intervention date should not differ systematically from the distribution of placebo effects. In other words, the placebo distribution serves as a benchmark against which the observed effect is contrasted.

The evaluation metric was the difference in mean error (DME) between the post-treatment and pre-treatment periods, computed for each placebo start date [
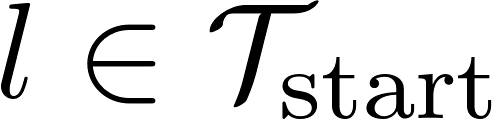
](https://www.codecogs.com/eqnedit.php?latex=l%20%5Cin%20%5Cmathcal%7BT%7D_%7B%5Ctext%7Bstart%7D%7D#0):

[
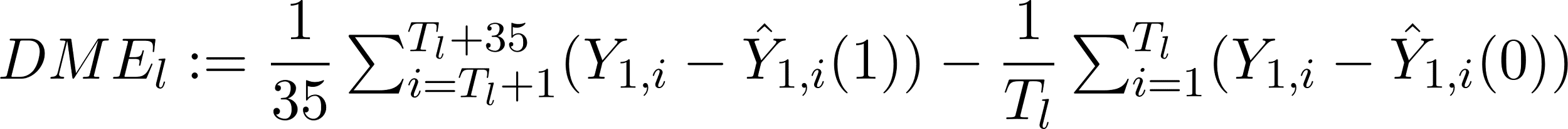
](https://www.codecogs.com/eqnedit.php?latex=DME_l%20%3A%3D%20%5Cdfrac%7B1%7D%7B35%7D%5Csum_%7Bi%3DT_l%2B1%7D%5E%7BT_l%2B35%7D%20%20(Y_%7B1%2Ci%7D-%5Chat%7BY%7D_%7B1%2Ci%7D(1))%20-%20%5Cdfrac%7B1%7D%7BT_l%7D%5Csum_%7Bi%3D1%7D%5E%7BT_l%7D%20(Y_%7B1%2Ci%7D-%5Chat%7BY%7D_%7B1%2Ci%7D(0))#0)

Because the intervention was expected to reduce outcomes, a left-sided Fisher-type test was applied, where more negative values of [
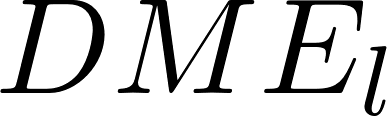
](https://www.codecogs.com/eqnedit.php?latex=DME_l#0) indicate stronger evidence of a treatment effect. The p-value was computed as:

[
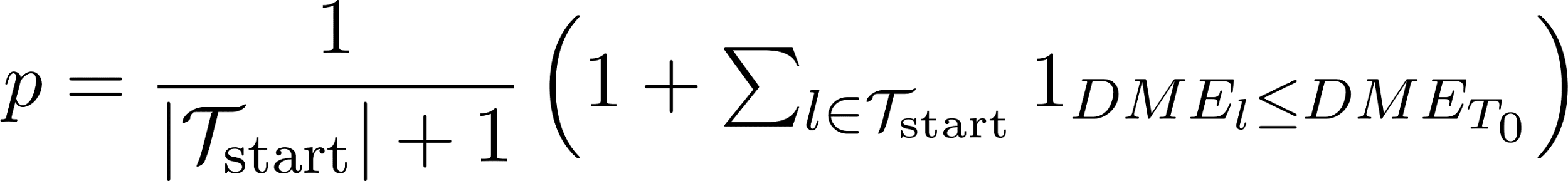
](https://www.codecogs.com/eqnedit.php?latex=p%20%3D%20%5Cdfrac%7B1%7D%7B%7C%5Cmathcal%7BT_%7B%5Ctext%7Bstart%7D%7D%7D%7C%2B1%7D%5Cleft(1%2B%5Csum_%7Bl%5Cin%5Cmathcal%7BT_%7B%5Ctext%7Bstart%7D%7D%7D%7D%201_%7BDME_l%20%5Cleq%20DME_%7BT_0%7D%7D%5Cright)#0)

where  is the statistic corresponding to the actual intervention date.

A small p-value indicates that the observed treatment effect was unusually large relative to the distribution of placebo effects, providing evidence against the null hypothesis of no effect. This inference procedure is valid under the assumption that treatment assignment is independent of both observed and unobserved predictors, namely:

This condition ensures that the untreated donor units provide a credible basis for constructing counterfactuals and that placebo distributions correctly approximate the sampling distribution under the null. In observational SCM applications, treatment assignment is not randomized. Instead, validity rests on the assumption that once observed covariates (, ) and latent factors (,) are accounted for, no remaining unobserved confounders simultaneously drive both treatment assignment and outcomes. In practice, this means that SCM does not require full randomization, but it does rely on the credibility of the model structure—particularly the ability of pre-intervention matching to capture both observed and unobserved drivers of outcomes. Placebo tests, goodness-of-fit diagnostics, and sensitivity analyses are therefore crucial for assessing whether this assumption is reasonable in the empirical context.

**Counterfactual Confidence Intervals**

Let  be the observed outcome for the treated unit and  the counterfactual predicted by ASCM. Let the cumulative treatment effect

For  we defined the residual as

for , where  represents the synthetic series estimated in the placebo test starting at week .

Let  be the calendar week of the year (ISO). Then, define  for each , i.e., the isocalendar weeks of the post-treatment period.

Let the set of placebo residuals observed in the same calendar week across all placebo test

For  we generate a seasonality sample of cumulative residuals

by drawing  from each set defined above uniformly with replacement.

Let  Under the assumption that placebo residuals from are exchangeable conditional of the iso week and independent through time, then we can construct the confidence intervals (CI) around  as the empirical percentiles 2·5% and 97·5% of .

Then, to construct the CI of total cost, where for each outcome  with unit cost , the cost impact is , and its CI applies the same shift.

Let  be the set of cumulative residuals in costs for each outcome. Under the assumption that cumulative residuals in cost are independent by outcome, we sample the total cumulative cost residuals as

for  and . We construct the CI around the empirical percentiles 2·5% and 97·5%.

**Seasonal and Catch-up Counterfactual Estimates Procedure**

Effectiveness Estimates

Because published estimates of nirsevimab effectiveness are available only for basic/intermediate (BM) and intensive care unit (ICU) outcomes, and these estimates are reported with relatively wide 95\% confidence intervals (CI) for each cohort group, they were treated as random variables and incorporated through simulation. The model assumes a hierarchical protection structure: effectiveness against medical attentions (MA) was considered equal to that against BM, while effectiveness against ICU outcomes was assumed to be higher.

To capture uncertainty, a Dirichlet distribution with three categories was calibrated for each cohort such that its simulated means and confidence intervals reproduced the published estimates. Specifically, given a parameter vector , samples were drawn as

and mapped to effectiveness values as

By repeatedly sampling from this distribution, the simulation propagated uncertainty in the effectiveness parameters into the counterfactual analysis. Dirichlet parameters were calibrated so that the simulated means and confidence intervals closely matched those reported by [2] (see Table S8).

| **Seasonal cohort effectiveness** | | | | |
| --- | --- | --- | --- | --- |
| **Event** | **Reported Mean** | **Reported CI 95%** | **Dirichlet Mean** | **Dirichlet CI 95%** |
| BM | 53·19% | (31·37% - 68·07%) | 53·20% | (32·56% - 73·44%) |
| ICU | 74·13% | (48·59% - 86·98%) | 74·07% | (53·92% - 89·99%) |
| **Catch-up cohort effectiveness** | | | | |
| **Event** | **Reported Mean** | **Reported CI 95%** | **Dirichlet Mean** | **Dirichlet CI 95%** |
| BM | 80·67% | (76·99% - 83·75%) | 80·67% | (75·25% - 85·62%) |
| ICU | 87·65% | (82·08% - 91·48%) | 87·65% | (83·07% - 91·65%) |

**Table S8.** Dirichlet distribution Calibration for nirsevimab effectiveness.

Cohort-Specific Counterfactual Estimates

To disentangle the contributions of the seasonal and catch-up cohorts to the counterfactual outcomes, a Monte Carlo simulation was implemented. Let  and  denote simulated effectiveness values for the catch-up and seasonal cohorts, respectively. With  indexing weeks and  representing the counterfactual number of cases at week , define:

- : coverage of the catch-up cohort at week ,
- : coverage of the seasonal cohort at week ,
- : size of the catch-up cohort population at week ,
- : size of the seasonal cohort population at week .

The at-risk population for catch-up and seasonal cohorts were then expressed as, respectively:

That is, the at-risk population includes both (i) individuals not yet covered and (ii) covered individuals not fully protected due to imperfect effectiveness.

The relative contribution of the catch-up group at week  was calculated as:

Assuming that , the expected number of counterfactual cases attributable to the catch-up cohort is:

Analogously, the expected number of counterfactual cases attributable to the seasonal cohort is:

Finally, the allocation procedure accounted for severity by applying a hierarchical structure: ICU outcomes were assigned first, followed by BM, and finally MA. At each step, cases assigned to a more severe category were subtracted from the available at-risk population before proceeding to the next category. This ensured consistency with the clinical trajectory of RSV disease and avoided double-counting across outcome levels.

**Total cost of the 2024 nirsevimab campaign**

The total cost of the 2024 Nirsevimab immunization program was calculated using a price of 310 USD per dose, corresponding to the mean price of nirsevimab, which was 297.500 CLP and already includes transportation and storage logistics.

We only accounted for the doses that were actually administered in the campaign, as unused doses were stored and used in 2025. A total of 72,841 doses were administered to the seasonal cohort and 72,246 to the catch-up cohort. The cost of the doses was 44.915.070 USD approximately. It was also considered a 5 USD cost for administering the doses to the catch-up cohort, accounting for 361.230 USD. This adds up to a total cost of the 2024 strategy 45.276.300 USD.

[ML] Number of medical leave days. [BM] number of bed-days in basic/medium beds

**Figure S3.** Generation of the synthetic series of days of medical leave (ML) with the three top weights assigned.

[MA] Number of medical attentions.

**Figure s4.** Generation of the synthetic series of medical attentions (MA) with the three top weights assigned.

[BM] number of bed-days in basic/medium beds

**Figure s5.** Generation of the synthetic series of basic and intermediate bed-days (BM) with the three top weights assigned.

[BM] number of bed-days in basic/medium beds

**Figure s6.** Generation of the synthetic series of ICU bed-days (ICU) with the three top weights assigned.

**Supplementary references**

[s1] Rubin D. Estimating causal effects of treatments in experimental and observational studies. ETS Research Bulletin Series 1972; 1972(2): i–31. <https://doi.org/10.1002/j.2333-8504.1972.tb00631.x>

[s2] Abadie A, Gardeazabal J. The economic costs of conflict: a case study of the Basque Country. Am Econ Rev 2003; 93(1): 113–32. https://doi.org/10.1257/000282803321455188

[s3] Abadie A. Using synthetic controls: feasibility, data requirements, and methodological aspects. J Econ Lit 2021; 59: 391–425. <https://doi.org/10.1257/jel.20191450>

[s4] Chen T, Guestrin C. XGBoost: a scalable tree boosting system. Proc 22nd ACM SIGKDD Int Conf Knowledge Discovery and Data Mining 2016; 785–94. <https://doi.org/10.1145/2939672.2939785>

[s5] Ferman B, Pinto C. Synthetic controls with imperfect pre-treatment fit. Quant Econ 2021; 12: 1197–1221. <https://doi.org/10.3982/QE1596>

[s6] Chernozhukov V, Chetverikov D, Demirer M, Duflo E, Hansen C, Newey W, Robins J. Double/debiased machine learning for treatment and structural parameters. Econom J 2018; 21: C1–68. https://doi.org/10.1111/ectj.12097

[s7] Abadie A. Comparative politics and the synthetic control method. *Am J Polit Sci* 2015; 59: 495–510. https://doi.org/10.1111/ajps.12116
